# Unique Hyperspectral Response Design Enabled by Periodic Surface Textures in Photodiodes

**DOI:** 10.1021/acsphotonics.4c00453

**Published:** 2024-06-07

**Authors:** Ahasan Ahamed, Amita Rawat, Lisa N. McPhillips, Ahmed S. Mayet, M. Saif Islam

**Affiliations:** Electrical and Computer Engineering, University of California—Davis, Davis, California 95616, United States

**Keywords:** avalanche photodiodes, hyperspectral imaging, multispectral imaging, photon-trapping features, spectral response engineering

## Abstract

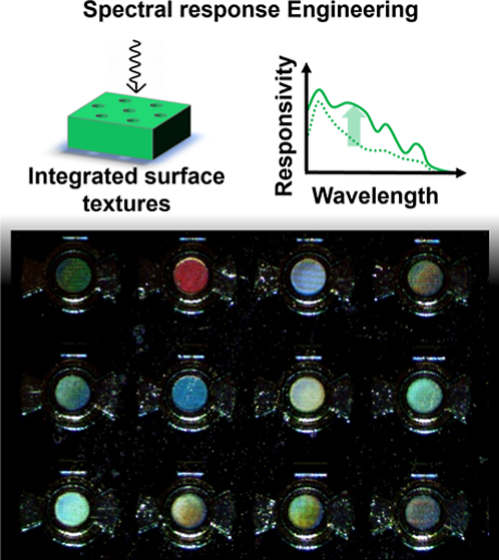

The applications of hyperspectral imaging across disciplines such as healthcare, automobiles, forensics, and astronomy are constrained by the requirement for intricate filters and dispersion lenses. By utilization of devices with engineered spectral responses and advanced signal processing techniques, the spectral imaging process can be made more approachable across various fields. We propose a spectral response design method employing photon-trapping surface textures (PTSTs), which eliminates the necessity for external diffraction optics and facilitates system miniaturization. We have developed an analytical model to calculate electromagnetic wave coupling using the effective refractive index of silicon in the presence of PTST. We have extensively validated the model against simulations and experimental data, ensuring the accuracy of our predictions. We observe a strong linear relationship between the peak coupling wavelength and the PTST period along with a moderate proportional relation to the PTST diameters. Additionally, we identify a significant correlation between inter-PTST spacing and wave propagation modes. The experimental validation of the model is conducted using PTST-equipped photodiodes fabricated through complementary metal-oxide-semiconductor-compatible processes. Further, we demonstrate the electrical and optical performance of these PTST-equipped photodiodes to show high speed (response time: 27 ps), high gain (multiplication gain, *M*: 90), and a low operating voltage (breakdown voltage: ∼ 8.0 V). Last, we utilize the distinctive response of the fabricated PTST-equipped photodiode to simulate hyperspectral imaging, providing a proof of principle. These findings are crucial for the progression of on-chip integration of high-performance spectrometers, guaranteeing real-time data manipulation, and cost-effective production of hyperspectral imaging systems.

## Introduction

Spectrometers find diverse applications in fields like astronomy, forensic science, art conservation, pharmaceutical, and molecular imaging, as well as environmental contamination monitoring.^1−8^ These applications have significantly influenced the miniaturization of spectrometers and the advancements in spectral response engineering methods. Fraunhofer et al., in 1898, CE^9^ showcased the first demonstration of prismatic and diffraction spectra in telescopic imaging. A large form factor spectrometers in hyper/multispectral imaging (HSI/MSI) have long been prevalent in astrophysics and military applications.^10,11^ Recently, these HSI and MSI technologies have found new applications in the analysis of biological tissues, molecules, and nonfunctional materials.^12^ In healthy versus infected tissues, the proteins such as hemoglobin and melanin exhibit unique interactions with electromagnetic (EM) waves. This necessitates an efficient and wavelength-resolved capture of reflected, fluorescent, and transmitted EM waves facilitated by HSI/MSI imaging technology. While Fourier transform infrared spectroscopy offers exceptional wavelength resolution and rapid response time,^13,14^ its bulky measurement setup and high power consumption hamper the scalability and accessibility of the system. Advances in fabrication technology have enabled miniaturized multispectral imaging and spectral response engineering.^15^ Several innovative approaches have been explored, including the use of broad-band diffractive and charge-coupled devices alongside a novel spectrum extraction algorithm,^16^ complementary metal oxide semiconductor (CMOS) circuitry enabling global shutters,^17^ evanescently coupled spiral waveguides on silicon-on-insulator (SOI) substrates with detector arrays,^18^ etalon arrays (two semireflecting surfaces separated by an optically transparent medium),^19^ photonic molecules with microring resonators,^20^ metasurface-based imaging,^21,22^ colloidal quantum dot imagers,^23,24^ inverse design for broad-band engineering,^25^ and surface gratings.^26^ Although miniaturized spectrometers and computational imaging systems have shown promises, challenges persist. Large device footprints, inefficient power consumption, complex peripheral circuitry, and the requirement for wavelength splitters and filters impede the further scalability of these systems.

**Figure 1:**
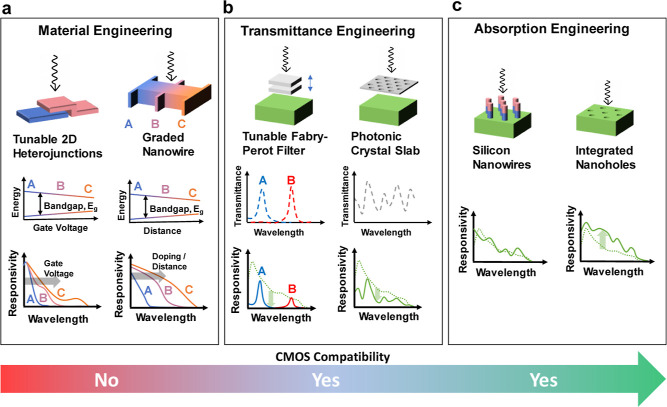
Various approaches used thus far for spectral response engineering: An illustration of spectral response engineering method (top) and their respective responsivity over wavelength (bottom). (a) Gradual bandgap modulation is enabled by gate voltage-controlled tunable two-dimensional (2D) material heterojunctions^39^ (left) and gradual doping^40^ or quantum scale width manipulation in the semiconductor^41^ material (right) allowing distinct responsivity profiles. These methods involve CMOS-incompatible exotic 2D material processing or complex growth processes. (b) Engineering transmittance of the devices by voltage-controlled thickness modulation of the Fabry–Pérot resonator filter^42^ (left) and photonic crystal slabs^43^ (right). This method is compatible with the CMOS technology, however results in reduced responsivity of the detectors. (c) Engineering absorption by using nanowire-based photodiodes^36^ (left) and integrated nanoholes in photodiodes^44^ (right). The introduction of nanostructures such as pillars or surface textures such as nano/microholes is CMOS compatible and uniquely enhances the light–matter interaction for each wavelength translating into unique responsivity.

Recent advances in spectrometer-on-chip technologies have explored engineered spectral responses in combination with artificial intelligence-assisted image reconstruction techniques.^27−29^ Various methods, including plasmonic surface structure-based spectral response modulation,^30^ bandgap engineering through alloying or superlattice stacking,^31^ transmission engineering,^32−34^ spectrum shaping using Si-perovskite and Ag nanowire-based hybrid detectors,^35^ nanowire array-based absorption engineering,^36^ nature-inspired organic photodetectors for MSI/HSI,^37,38^ and metasurfaces,^22^ have demonstrated decent spectral resolution. A compilation of available spectral response engineering approaches is presented in Figure 1. However, integrating these methods into the CMOS process line faces challenges due to complex fabrication processes, the use of exotic materials, and the adaptation of nonscalable device designs. Consequently, there is a growing demand for monolithic CMOS-compatible, cost-effective, and reliable hyperspectral sensors with a unique responsivity.

The integration of photon-trapping surface texture (PTST) into photodiodes has been thoroughly explored to enhance EM wave absorption and control light–matter interactions for high-speed sensing.^44−52^ However, their potential in spectral response design remains unexplored, primarily due to the lack of a comprehensive analytical framework. In this study, we highlight the use of PTST in molding the absorption spectral response of photodiodes. We introduce a comprehensive analytical framework for designing PTST tailored to the desired spectra, enabling customization of absorption spectra through the manipulation of PT hole diameter (*d*) and periodicity (*p*). We fabricated various PTST-equipped photodiodes that exhibit distinct absorption spectra represented by external quantum efficiency (EQE) values. Finally, we validate our analytical predictions against experimental and Lumerical FDTD simulation results. Such frameworks can help designers to accurately customize spectra and estimate spectral responses. This analytical model not only assists in creating succinct SPICE models for PTST-equipped photodiodes but also offers pedagogical insights and an intuitive understanding of EM wave interactions in PTST-equipped devices.

## Methodology

### Formulation of the Coupling Coefficient

Accurate spectral response engineering requires an intuitive understanding of light–matter interactions based on physics principles. In this section, we introduce an analytical formulation describing the interaction between EM waves and the introduced PTST holes, represented by the coupling coefficient, κ.^53−55^ The coupling behavior of EM waves with different PTST dimensions is validated through experiments and simulations. The details of the simulation setup are available in the Supporting Information (Figures S1 and S2).

**Figure 2:**
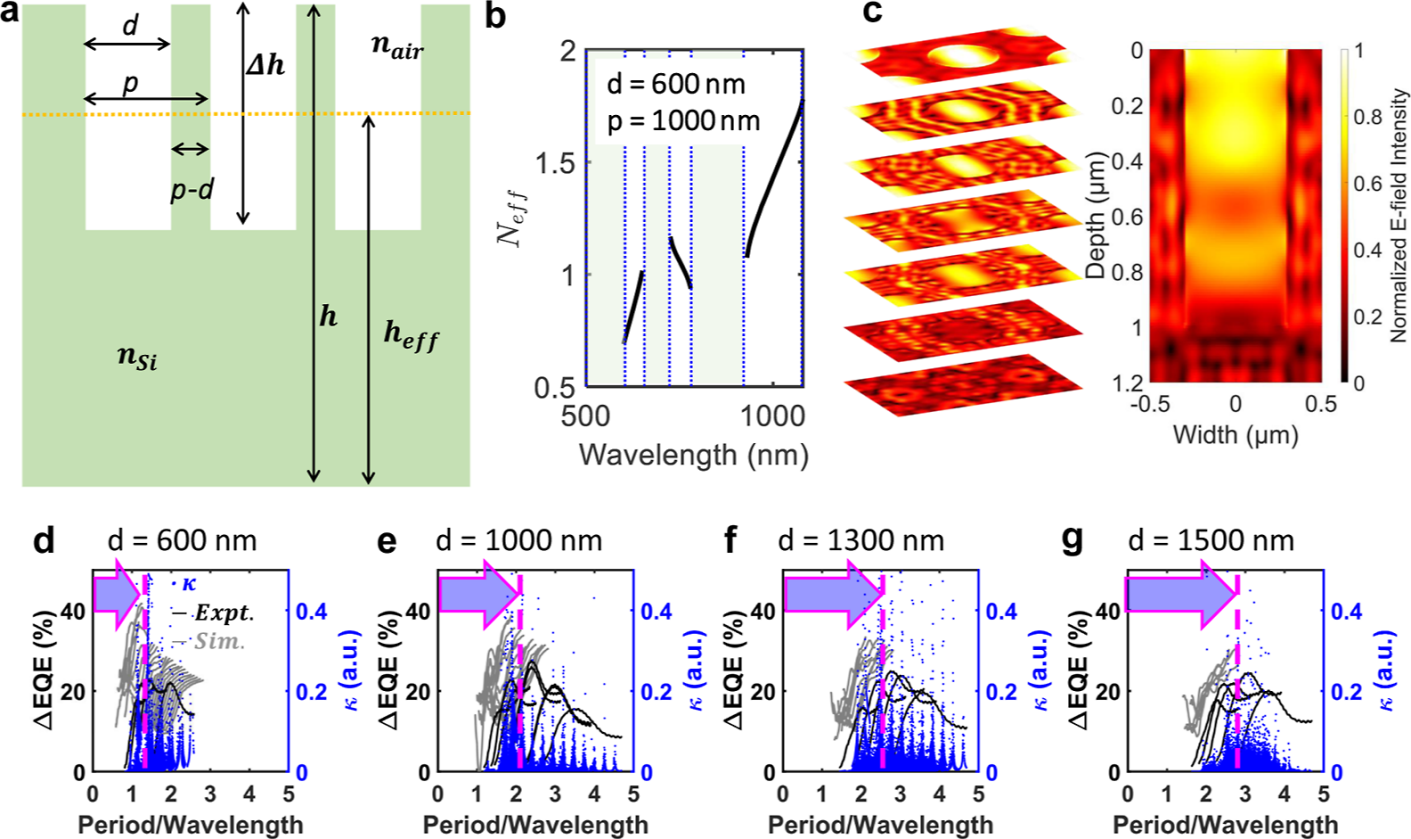
Mathematical formulation of the light–matter interaction: (a) simplified 2D cross-section of PTST used for the analytical model; (b) effective refractive index (*N*_eff_) for *d* = 600 nm, *p* = 1000 nm, and Δ*h* = 1000 nm PTST arrangement. The voids in *N*_eff_ represent photonic bandgap formation; (c) TE mode propagation profile shown for a unit cell of the PTST array. (d–g) Comparison of experimentally measured external absorption efficiency (i.e., EQE in black), simulated internal absorption efficiency (i.e., IQE in gray) on the FDTD Lumerical platform, and analytically calculated coupling coefficient (κ in blue) as a function of PTST period and illumination wavelengths for (d) 600, (e) 1000, (f) 1200, and (g) 1500 nm PTST diameters. The *p*/λ concerning the peak absorption efficiency and coupling increases as the diameter of the PTST increases from 600 to 1500 nm. For smaller PTST diameters, the PTST period comparable to the illumination wavelength result in maximum coupling and absorption efficiency, whereas for larger PTST diameters, the shorter EM wavelengths show better coupling.

In Figure 2a, we present a simplified 2D cross-section of the PTST array introduced into Si to build an analytical model for EM wave coupling. Figure 2c shows the trans electric (TE) mode propagation in a PTST-equipped device simulated in the Lumerical FDTD platform.
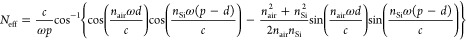
1

Equation 1 presents an analytical formulation of the effective refractive index (*N*_eff_) of Si after introducing the PTST,^54^ where ω = 2π/λ is the angular frequency, *c* is the speed of light in free space, *d* and *p* are the PTST diameter and periodicity, respectively, and *n*_air_ and *n*_Si_ are the refractive indexes of air and Si, respectively. Figure 2b shows the *N*_eff_ calculated for a fixed PTST dimension (diameter, *d*: 600 nm; period, *p*: 900 nm) as a function of ω*p*/*c*. The discontinuity (presence of voids) in the *N*_eff_ trend represents the photonic bandgap formation with the introduction of PTST that selectively forbids the EM wave coupling. A real *N*_eff_ results in a strong EM wave coupling, whereas the presence of the photonic bandgap results in a weak intrinsic absorption (weak EM wave coupling) governed by the electronic bandgap of the material.
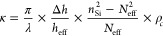
2where
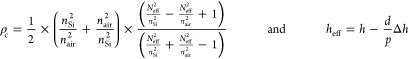


Further, using eq 1, the expression for coupling coefficient, κ, is formulated in eq 2, where ρ_c_ is the reduction factor and *h*_eff_ is the effective height of silicon as shown in Figure 2a.

The proposed analytical model is limited to calculating the coupling coefficient of the EM waves and is only proportionately related to the EQE. The coupling coefficient κ is one of the factors defining the EQE, along with the intrinsic material properties, electrical response, and structural properties, of a silicon-on-insulator substrate.

### TE Model Dispersion Relation


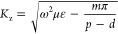
3In the presence of a PTST array in the photodiode, the TE and transmagnetic mode propagation is limited by the width of the *p* – *d* region as shown in Figure 2a. In this work, we have studied only the TE mode propagation and its impact on the EQE. The EM wave is introduced in the Si absorber layer and propagates through the *p* – *d* region. The TE mode dispersion relation is given by eq 3, where μ and ϵ are the magnetic permeability and electric permittivity of silicon and *m* is an integer.

## Results

We engineered the absorption spectral response by incorporating PTST into the photodiodes. The devices are fabricated by using CMOS-compatible processes on an SOI wafer. The die consists of various PTST dimensions, where the PT hole diameter range varies from 600 to 1500 nm and the period range varies from 900 to 3000 nm. The PT hole depth of 1000 nm is utilized to maximize the coupling for a broad range of wavelengths into the absorbing layer. The details for the device fabrication process steps are presented in the Supporting Information (Figure S3).

**Figure 3:**
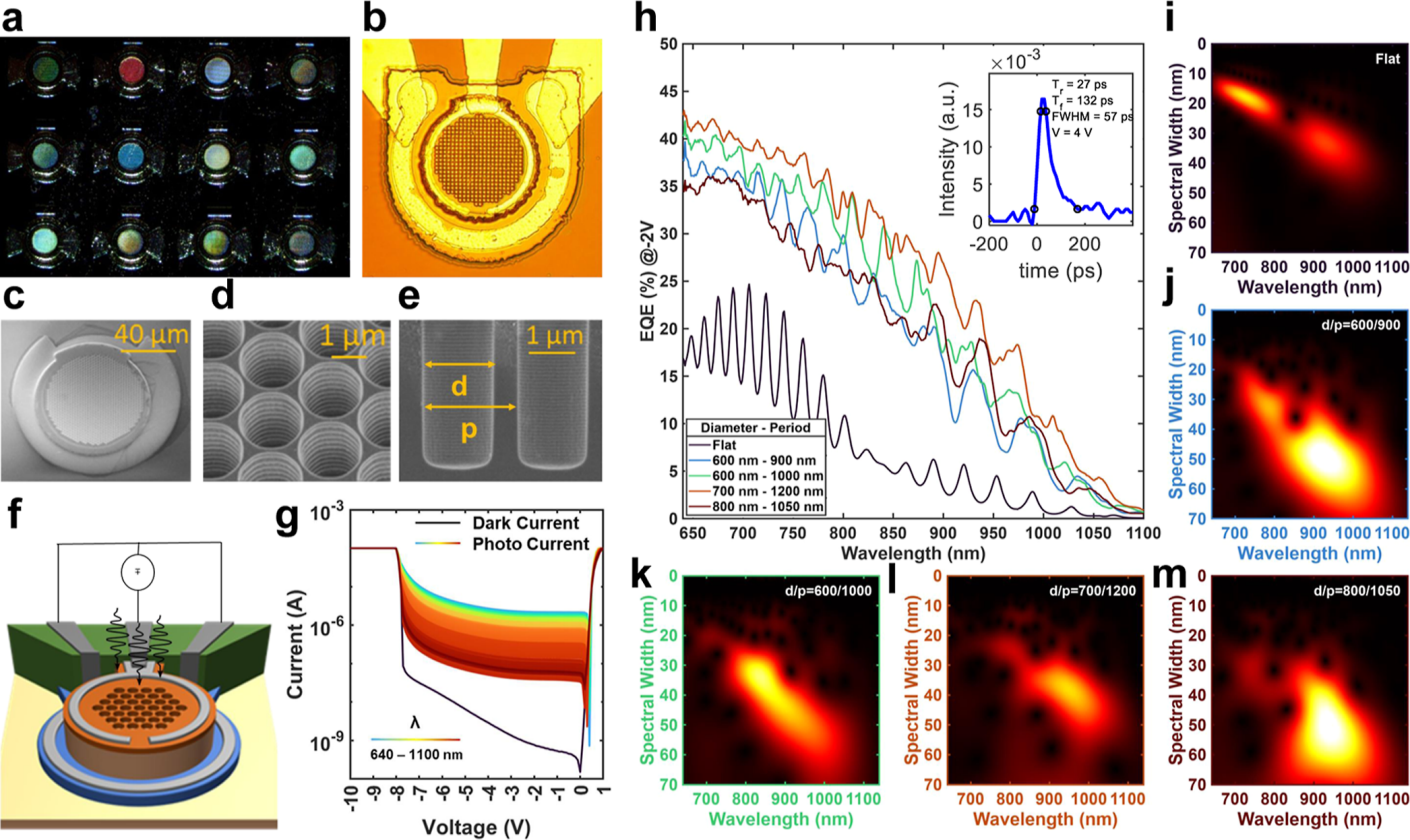
Fabricated photodiodes with unique spectral response: (a,b) Optical micrograph of the fabricated photodiodes with PTST features showcasing iridescence under white light evincing their unique responsivity. (c–e) SEM images of the device and the introduced PTST features. The use of ICPRIE has resulted in smooth and straight PTST sidewalls essential to minimize surface scattering. (f) Schematic depiction of the device fabricated and applied electrical and optical stimuli. (g) DC *I*–*V* characteristics of the photodiode are in the dark and under the illumination of laser wavelengths ranging from 640 to 1100 nm. (h) Unique EQEs of four photodiodes with PTST are compared against that of a flat device for a range of illumination wavelengths at a fixed laser power of 10 μW and an applied bias of −2 V. The legend depicts the PTST hole diameter and period. The EQE of the flat device shows resonance due to the Fabry–Pérot cavity effect, while the devices with PTST show enhanced EQE with unique spectral responses. The inset shows the transient response of a photodiode with device diameter, 20 μm. (i–m) Corresponding Morlet wavelet transforms (color-coded axes) for each photodiode showcasing their unique responsivity. The *x*-axis plots illumination wavelengths, while the *y*-axis depicts the spectral width of the respective crest and trough. The subtle uniqueness in the 2D EQE spectral response is prominently evident in the Morlet wavelet transforms.

The devices exhibit iridescent colors due to varying PTST dimensions, as shown in the microscopic images in Figure 3a,b. In Figure 3c,e, we show scanning electron microscopy (SEM) images of PTST. The line-of-sight etching process enabled by inductively coupled reactive ion etching results in straight side walls that help reduce the surface scattering of the EM waves. Figure 3f showcases the device characterization bias arrangement. A semiconductor parameter analyzer is used to apply a direct-current (DC) bias voltage ranging from 0 to 1 V in the forward bias and 0–10 V in the reverse bias. *NKT* upper continuum laser source and a wavelength filter are used to vary the illumination wavelength. The captured DC current–voltage (*I*–*V*) profile for a range of illumination wavelengths (640–1100 nm) at a fixed illumination power of 10 μW is shown in Figure 3g. The *I*–*V* trends under illumination are compared with the dark *I*–*V* profile. Figure 3h presents the EQE trends measured from four different PTST-equipped photodiodes and compared against that of a flat (without PTST) photodiode. We observe a consistent increase in the EQE for all the tested devices compared to the flat devices, owing to the photon-trapping technique.^44,45,52^ The EQE of a flat device possesses regular fringes as an outcome of Fabry–Pérot cavity resonance.^44^ Additionally, each device with PTST exhibits a distinct EQE response attributed to unique EM wave interactions with various PTST feature sizes. The inset in Figure 3h illustrates the transient behavior of a photodiode with a device diameter of 20 μm, demonstrating a response time of 27 ps. Additionally, the Supporting Information (Figure S4) provides the response time for a photodiode with a device diameter of 30 μm and an associated multiplication gain of 90 units. In Figure 3i–m, we showcase the Morlet wavelet transform of the EQE profile to highlight the influence of PTST on the absorption of the illumination wavelength and corresponding spectral width, resulting in their corresponding EQE trend. The apparent uniqueness in the EQE trends of various PTST-equipped photodiodes shown in Figure 3h has been made prominent in their respective wavelet transform contour plots. Additionally, we have experimentally demonstrated the influence of PTST density within the device on the EQE and showcased the manipulation of Fabry–Pérot resonance with varying *p* at a fixed *d* in Figure S5 (Supporting Information).

The EQE trend modulation with the introduction of the PTST array into the photodiodes is an outcome of a unique EM wave interaction for a range of PTST diameter, *d*, and periodicity *p* variations. The *d* and *p* are comparable to the illumination wavelengths which result in photonic bandgap formation and a nonuniform absorption of the EM wave.^54^ In Figure 2d–g, we analytically calculate the EM wave coupling coefficient (κ) for a range of PTST dimensions and illumination wavelengths and present a good match against the experimental and simulated ΔEQE [ΔEQE = EQE – mean (EQE)] trend. The experimental data are collected from 42 distinct photodiodes, encompassing a variety of *p* and *d* values. We show that a wider PTST diameter requires a larger PTST period per unit wavelength for better coupling, i.e., for a fixed PTST period, a denser PTST array will enable stronger coupling for shorter wavelengths. Such controlled interaction of EM waves enabled by PTST facilitates a framework for spectral response engineering.

## Discussion

### Coupling Analysis

**Figure 4:**
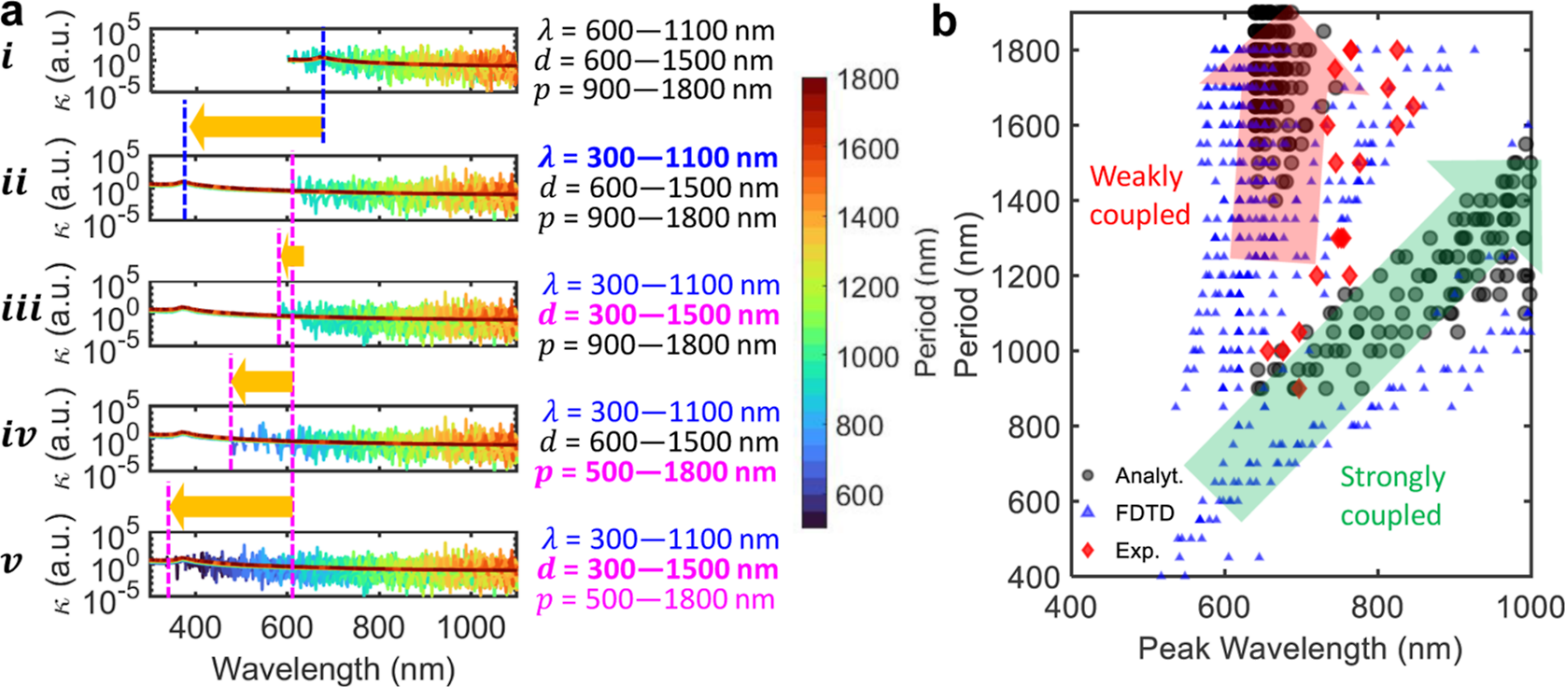
Coupling and fringes: (a) Coupling coefficient versus wavelength trends compared against five different combinations of λ, *d*, and *p* range from (i) to (v). In (i), κ is plotted against wavelength for a range of λ, *d*, and *p* aligned with that of experiments. The presence of a solid monotonous trend of weakly coupled scenarios overlapped with strongly coupled spikes showcasing the dual coupling nature. Changing the range of λ to 300–1100 nm in (ii) only enables the lower wavelengths to exhibit a weak intrinsic absorption-driven coupling, whereas the strong coupling remains intact as the *d* and *p* range. Further changing the range of *d* in (iii) marginally enables a strong coupling at lower wavelengths, and changing the range of *p* to 500–1800 nm results in a significant increase in the strong coupling at lower wavelengths in (iv). Finally, changing the range of both *d* and *p* to 300–1500 nm and 500–1800 nm, respectively, allows a strong coupling to occur even at further lower wavelengths in (v). (b) Comparison of peak coupling wavelength versus PTST period among experimental, FDTD simulation, and analytical trends. Analytical formulation suggests a linear relationship of the peak wavelength with the PTST period which is also reflected in simulation and experimental data (the green guiding arrow). The analytically predicted weakly coupled scenarios are evident in the experimental and FDTD simulation results (the red guiding arrow).

Due to the PTST-governed photonic bandgap formation, there exist weakly coupled and strongly coupled light–matter interaction scenarios.^56^ The weakly coupled scenario follows the intrinsic absorption characteristics of the material (i.e., silicon), whereas the strongly coupled scenarios exhibit PTST-dependent EM wave coupling in addition to the intrinsic absorption. In Figure 4a, we have plotted the coupling coefficient calculated from eq 2 by changing the range of λ, *d*, and *p*. We show that the κ exhibits the aforementioned coupling scenarios, a weak intrinsic absorption-driven coupling plotted in a solid monotonous trend, and strongly coupled scenarios plotted as overlapping spikes in Figure 4a. The series of figures from (i) to (v) present the weak and the strong coupling behavior by manipulating the range of λ, *d*, and *p*. Figure 4ai represents the baseline κ profile in alignment with the experiments, i.e., the range of λ, *d*, and *p* is 600–1100, 600–1500, and 900–1800 nm, respectively. In Figure 4aii, we have reduced the lower bound of λ to 300 nm while keeping the *d* and *p* range fixed. Doing so only impacts the weakly coupled scenarios while keeping the strongly coupled scenarios intact. This shows that the strongly coupled scenarios are strictly governed by PTST dimensions, i.e., *d* and *p*. In Figure 4aiii, we have reduced the lower bound of *d* to 300 nm while keeping the range of λ and *p* consistent with Figure 4aii. The introduction of lower *d* values marginally allows lower wavelengths to undergo strong coupling. Further, we reduced the lower bound of *p* to 500 nm while keeping the range of λ and *d* consistent with Figure 4aii. This further allows significantly lower wavelengths to undergo strong coupling, as shown in Figure 4aiv. Finally, we reduced the lower bounds of both *d* and *p* to 300 and 500 nm, which results in a strong coupling throughout the wavelength range (Figure 4av). This exercise concludes that the weakly coupled wavelengths are purely material dependent and governed by the intrinsic absorption coefficient. The strongly coupled scenarios are governed by the interplay of *d* and *p* with a strong dependence on the *p*. To establish a qualitative relationship between the EM wave coupling and PTST dimensions, we extracted the wavelengths at which the peak coupling occurs for a range of *d* and *p*. The extracted peak coupling wavelengths exhibit two distinct slopes representing weak and strong coupling, as shown in Figure 4b. The weakly coupled peak wavelengths are bounded by the lower bound of the wavelength range used in the analysis. The peak wavelengths in the strongly coupled scenario exhibit a fairly linear relationship with the period, *p*. Further detail of the analysis is presented in the Supporting Information (Figures S6 and S7). Furthermore, we have validated the analytically generated period vs peak wavelength trend with FDTD simulations and experimental results. We observe the presence of weakly coupled and strongly coupled scenarios in the simulations and experiments as predicted by the analytical model as shown in Figure 4b.

### TE Mode Analysis

We have fabricated PTST-equipped photodiodes that are shown to have unique EQE profiles with PTST-dependent fringes in the EQE versus the wavelength spectrum. We have shown that for a given PTST array with diameter, *d*, and period, *p*, arranged in a hexagonal lattice pattern, the fringes are defined by the (*p* – *d*) region (the narrowest most silicon region).

**Figure 5:**
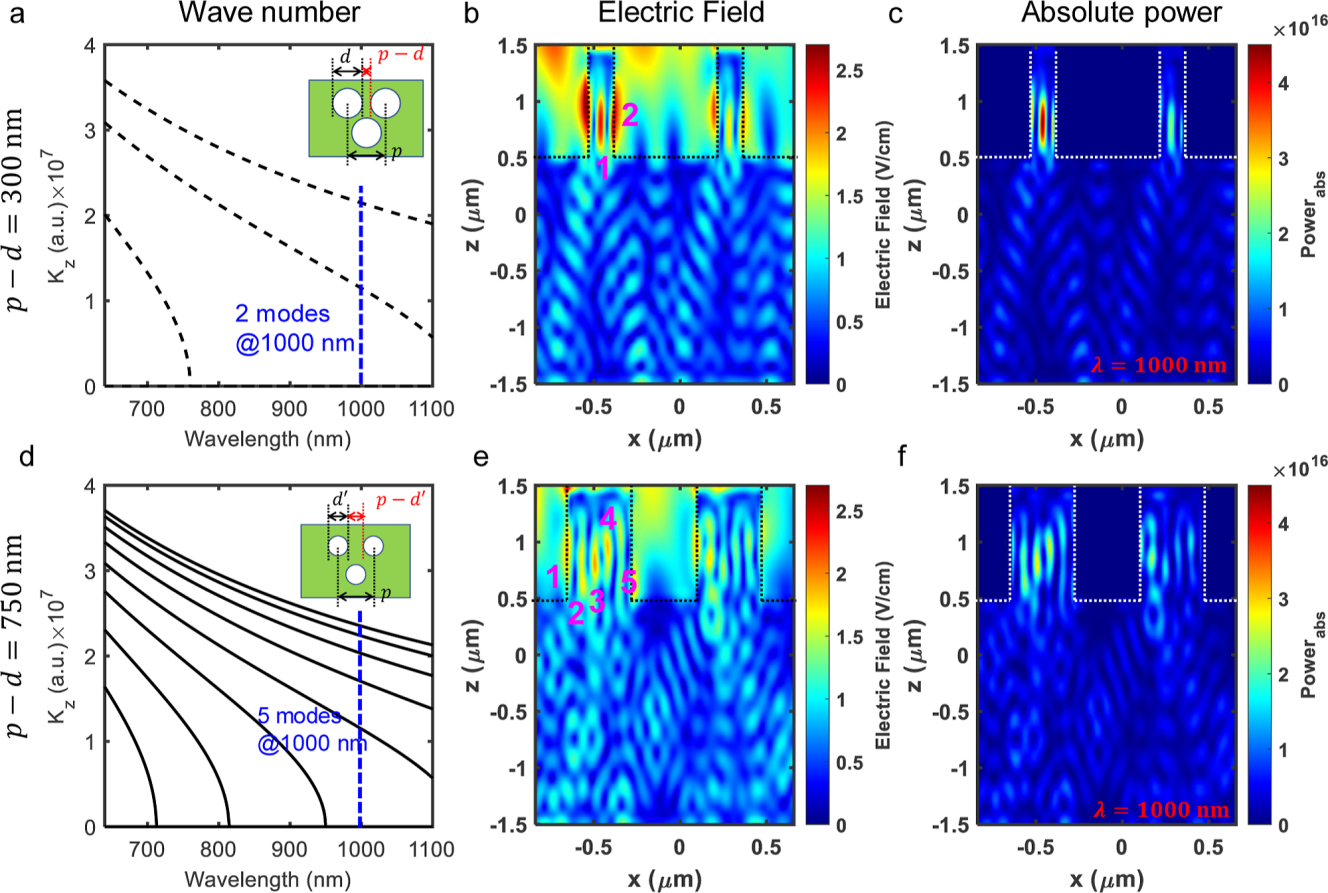
Feasible TE modes for a given *p* and *d*: (a) wave number, *K*_z_, in the direction of EM wave propagation as a function of illuminated wavelength (dispersion curve) highlighting feasible TE modes for smaller *p* – *d* = 300 nm. The blue dotted line shows that at 1000 nm illumination wavelength, only two TE modes are feasible. (b,c) Electric field profile that propagates at 1000 nm illumination wavelength and corresponding power absorption showcases the presence of two modes. (d) Dispersion curve for *p* – *d* = 750 nm showing five possible TE modes at 1000 nm wavelength. (e,f) Electric field propagating through the *p* – *d* region and corresponding power absorption showcasing five TE modes.

Figure 5a shows the dispersion trend as a function of illumination wavelength for a small *p* – *d* = 300 nm. We show that only a few TE modes are feasible in the wavelength range of interest. We highlight that at a fixed wavelength of 1000 nm, only two TE modes are feasible. In Figure 5b,c, we plotted the electric field propagation profile and respective power absorption to highlight the presence of two modes.

Figure 5d shows the dispersion trend as a function of illumination wavelength for a large *p* – *d* = 750 nm. We show that plenty of TE modes are feasible in the wavelength range of interest. We highlight that at a fixed wavelength of 1000 nm, five TE modes are feasible. In Figure 5e,f, we plotted the electric field propagation profile and respective power absorption to highlight the presence of five modes. The presence of multiple modes per wavelength results in diluted fringes in the EQE profile.

## Proof-of-Principle Demonstration

**Figure 6:**
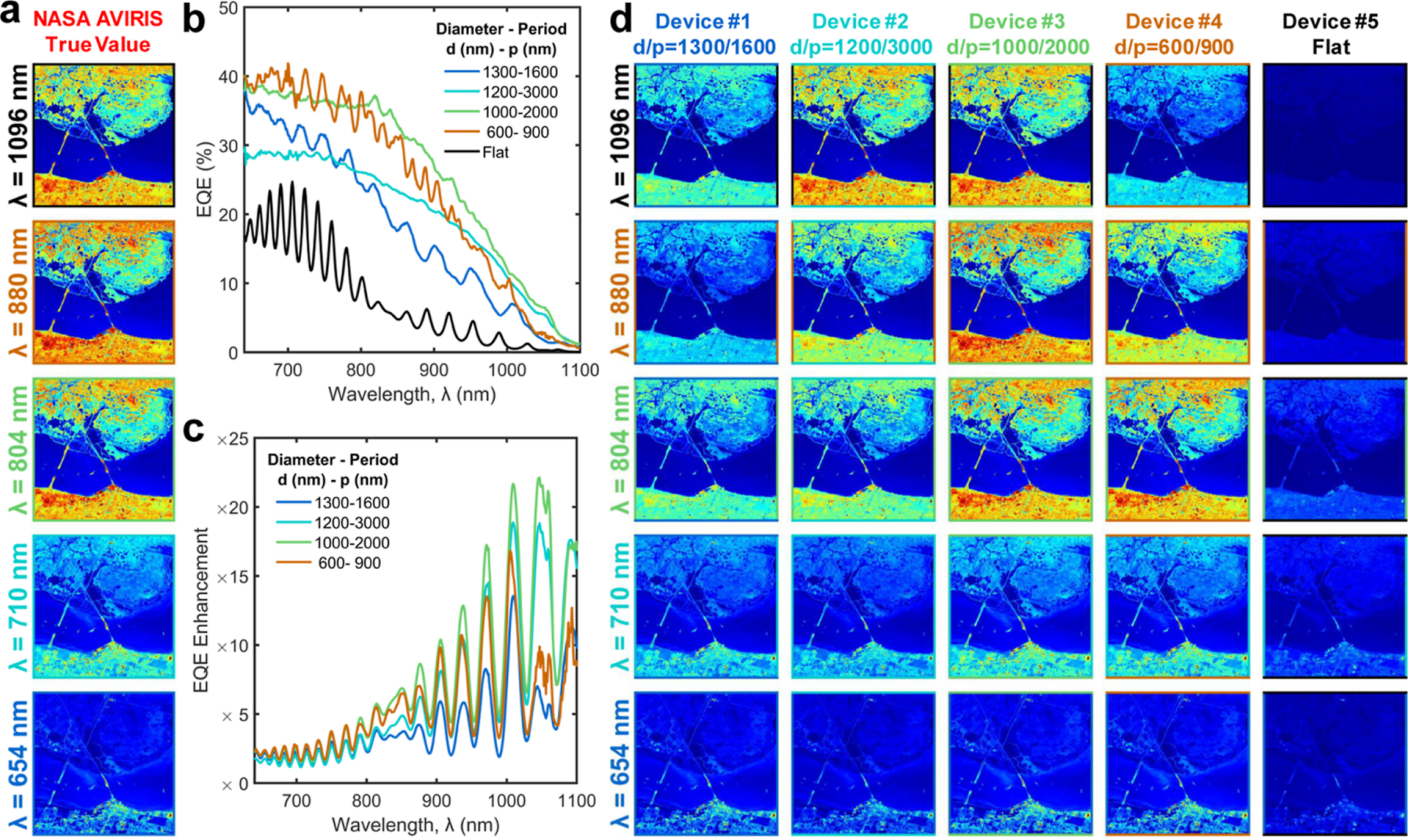
Proof-of-principle demonstration: (a) HSI of Kennedy Space Center captured by NASA AVIRIS is shown at wavelengths 654, 710, 804, 880, and 1096 nm. (b) EQE and (c) ΔEQE comparison among silicon photodiodes with integrated PTST with varying diameters, *d* and periods, *p*, and a flat device. (d) Simulated HSI image prediction of Kennedy Space Station for the same wavelengths (654, 710, 804, 880, and 1096 nm) using four unique PTST-equipped devices and a flat device. The PTST-enabled wavelength selective imaging allows efficient capture of finer details of the frame on a silicon platform.

To demonstrate the application of these unique response photodetectors, we have used the HSI data set captured for the Kennedy Space Center (KSC) by NASA AVIRIS^57,58^ and unit device performance to predict the image sensor array performance. The image sensors used in AVIRIS are InSb-based photodiodes. We have chosen 654, 710, 804, and 1096 nm wavelength images captured by AVIRIS, and false color images at each wavelength are shown in Figure 6a. In this study, we predict the image formation at each wavelength using Si-based flat and PTST-equipped photodetectors. Comparison of the EQE and ΔEQE trends for flat (without PTST) and PTST-equipped devices is shown in Figure 6b,c. The EQE responses presented in Figure 6b were utilized in predicting the photocurrent generated by each detector. The introduction of the PTST array into the photodetector increases the EQE over the entire range of wavelength spectra and results in a greater contrast as against the flat photodetector, as shown in Figure 6d. The images captured using flat photodetectors exhibit faint contrast compared to the PTST-equipped detectors enabling CMOS-compatible near-infrared hyperspectral imaging capabilities that AI-assisted image processing algorithms can further enhance. Also, the variation in PTST provides unique responses across different devices. It is clearly illustrated in Figure 6d, where we can observe a better response at longer wavelengths in device #2 and shorter wavelengths in device #4. In contrast, device #3 performs optimally in a broad range of wavelengths. Reducing the correlation between spectral responses among different devices is important for achieving high reconstruction accuracy and robust noise tolerance. We will implement strategies to introduce significantly dissimilar device variations in future designs.

Last, we have presented the details of the spectral reconstruction scheme using linear approximation methods to show the reconstruction efficiency ranging from 1.49 to 10.97% depending on the ground truth (Figure S9). Details of the reconstruction scheme are mentioned in the Supporting Information (section: Spectral Reconstruction Scheme).

### Performance Benchmarking

We present a thorough device performance benchmarking based on the device form factor, wavelength range covered, and compatibility with existing CMOS processes in Table 1. Most of these outstanding spectrometer approaches are not compatible with the CMOS processes, and a few CMOS-compatible approaches require a larger form factor. The devices with engineered spectral responses proposed in this work show a wide wavelength range coverage and a smaller device footprint, and the device fabrication processes used are CMOS-compatible..

**Table 1:** Benchmarking Table Comparing the Device Performance against the Existing Literature

ref.	materials used	wavelength range (nm)	form factor (μm^2^)	CMOS compatibility
2014^16^	polychromate diffractive optics with CMOS sensors	300–2500		not compatible
2016^18^	evanescently coupled multimode Si spiral waveguide	1520–1522	π250^2^	compatible
2017^19^	Fabry–Pérot etalons	400–900	500 × 500	not compatible
2019^43^	photonic crystal slab on CMOS sensors	550–750	32 × 32	compatible
2019^36^	silicon nanowire array	400–800	150 × 150	compatible
2020^59^	perovskite quantum dot filters and CCD	250–110	7 × 10^4^ × 7 × 10^4^	not compatible
2021^60^	single tunable black phosphorus detectors	2000–9000	9 × 16	not compatible
2021^61^	plasmonic nanohole array on glass with CMOS sensors	480–750	100 × 100	
2022^62^	MoS_2_/WSe_2_	405–845	22 × 8	not compatible
2022^24^	PbS colloidal quantum dots	400–1300	15 × 15	not compatible
2022^39^	ReS_2_/Au/WSe_2_ heterojunction	1150–1470	∼20 × 20	not compatible
2023^20^	photonic molecule	1500–1600	60 × 60	compatible
this work	photon trapping microholes in Si	640–1100	π10^2^	compatible

## Conclusions

We present the design and fabrication of efficient, high-speed, high-gain photodiodes with unique spectral responses by introducing PTST. We present an analytical formulation of EM wavelength coupling as a function of the PTST dimensions. We show that the peak coupling wavelength exhibits a linear relationship with the PTST period, *p*, and a weak dependence on the PTST diameter, *d*. We also show that the *p* – *d* value controls the extent of fringes in the absorption efficiency of the device. These PTST-equipped photodiodes and their analytically explained EM wave interaction can potentially transform the spectral response engineering. The unique spectral responses through PTST incorporation will enable computational imaging, along with an opportunity for extreme miniaturization and on-chip integration of spectrometers, facilitating a pivotal step forward in realizing the on-chip high-performance hyperspectral imaging systems.

## Supporting Information Available

Details of device fabrication, characterization, and simulations The data can be made available on request (ZIP)
PTST-equipped device structure simulated in FDTD; optical absorption efficiency; fabrication process flow; transient response; Fabry–Pérot effect modulation; EM wave coupling analysis; manipulation of EQE fringes through control of TE mode propagation; measured unique absorption spectra; and reconstruction of spectral response spectrum (PDF)
