# Supplementary figures and images for: Unique Hyperspectral Response Design Enabled by Periodic Surface Textures in Photodiodes
Source: ACS Photonics. 2024 Jun 7;11(6):2497–505. doi: 10.1021/acsphotonics.4c00453 (PMC11191742; doi:10.1021/acsphotonics.4c00453)

**a****Flat**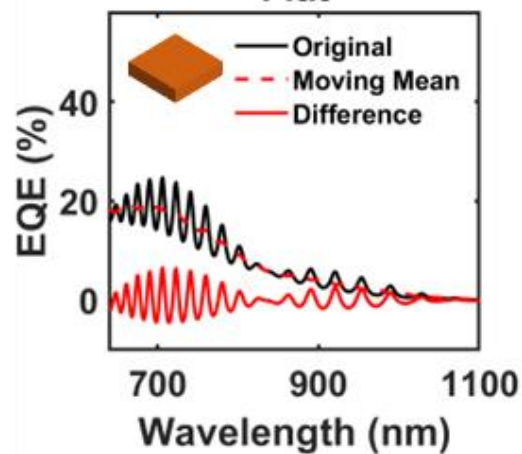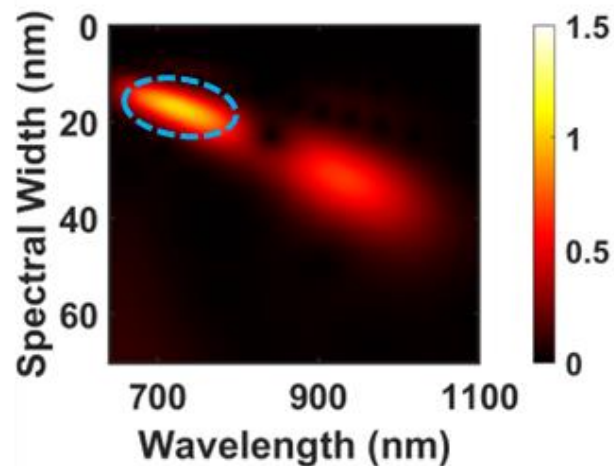**b****Diameter - Period  
1000 nm - 3000 nm**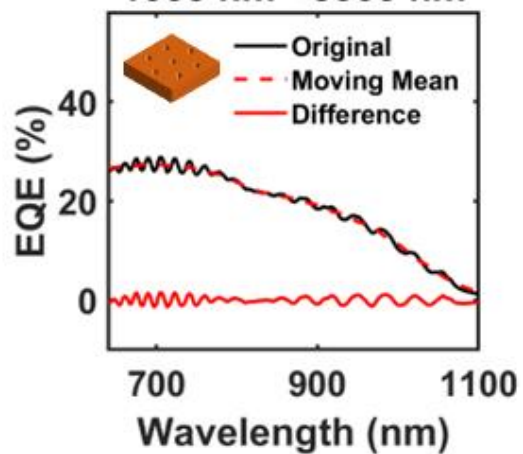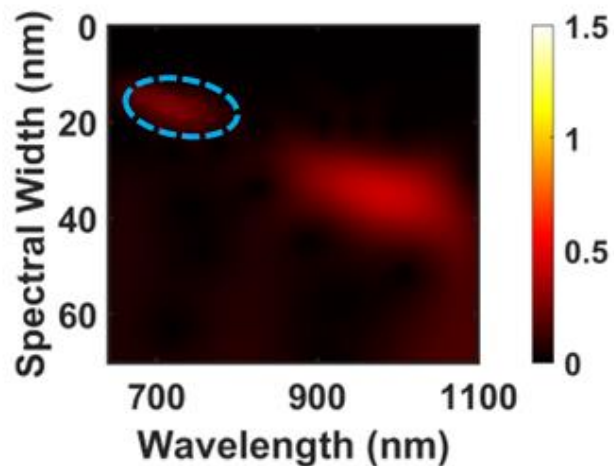**c****Diameter - Period  
1000 nm - 2500 nm**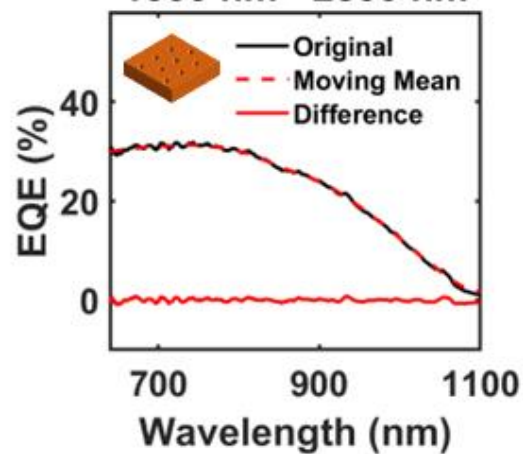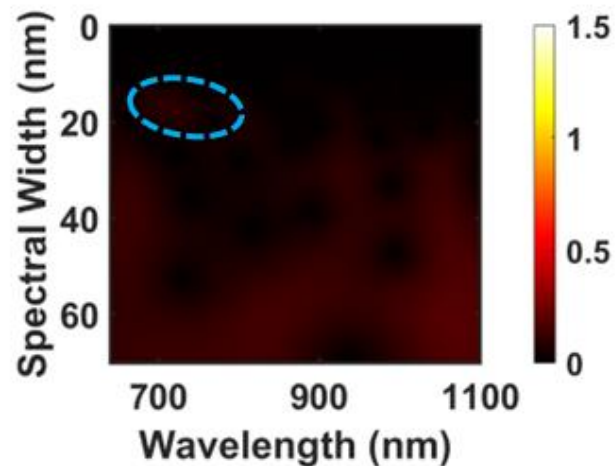**d****Diameter - Period  
1000 nm - 2000 nm**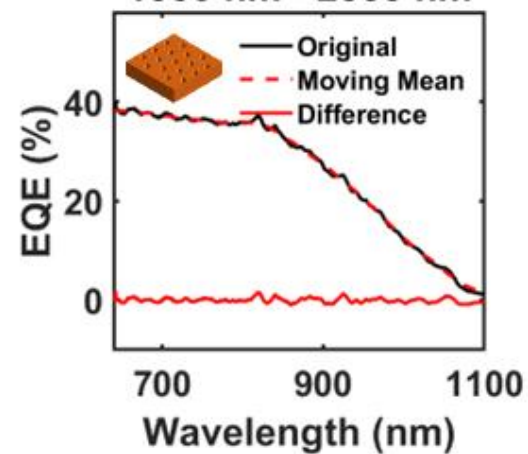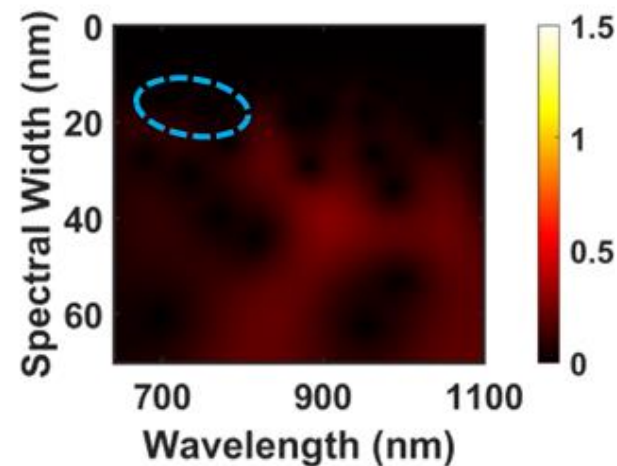

Supplement: Supplementary file 1 — ph4c00453_si_001.zip [file ph4c00453_si_001.zip › Vertical_resonance_modulation.pdf]

**a**

$\lambda$ : 600-1100 nm  
d: 600-1500 nm  
p: 900:3000 nm

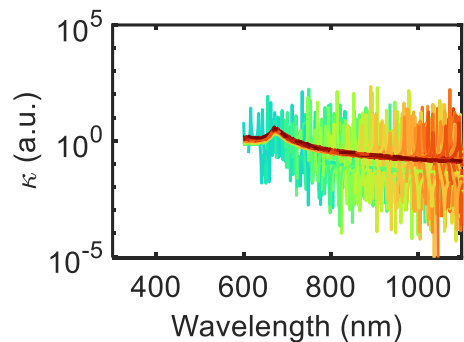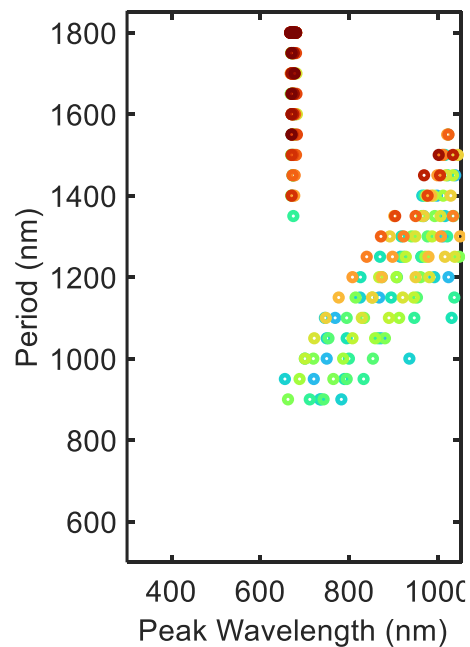**b**

$\lambda$ : **300-1100 nm**  
d: 600-1500 nm  
p: 900:3000 nm

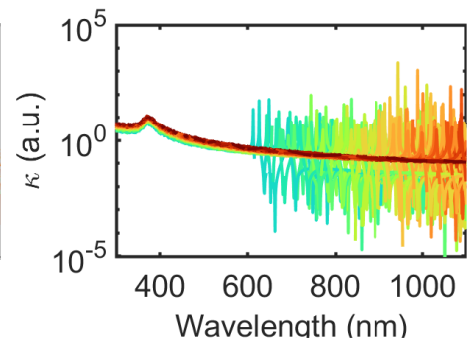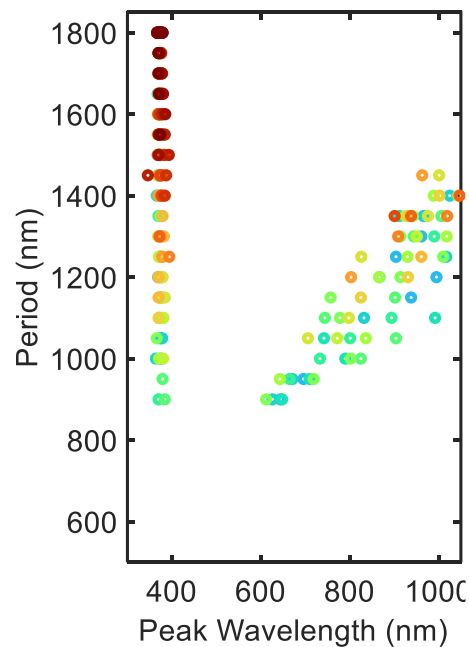**c**

$\lambda$ : 300-1100 nm  
d: **300-1500 nm**  
p: 900:3000 nm

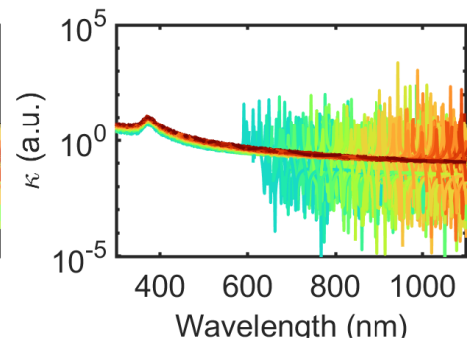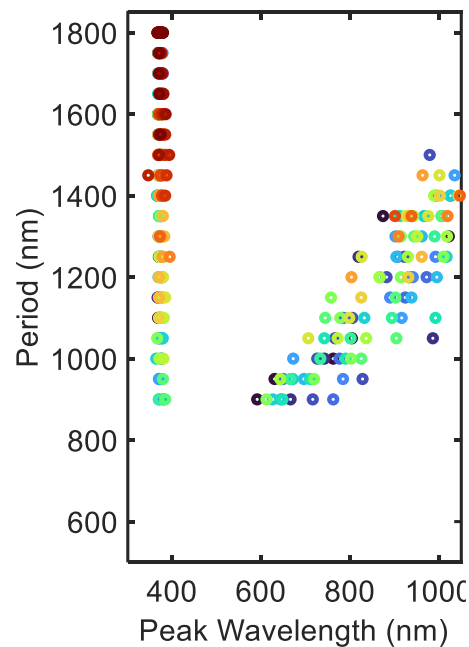**d**

$\lambda$ : 300-1100 nm  
d: 600-1500 nm  
p: **500:3000 nm**

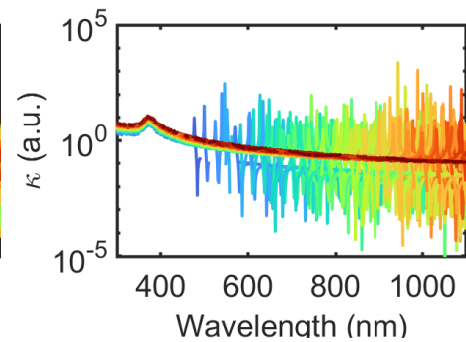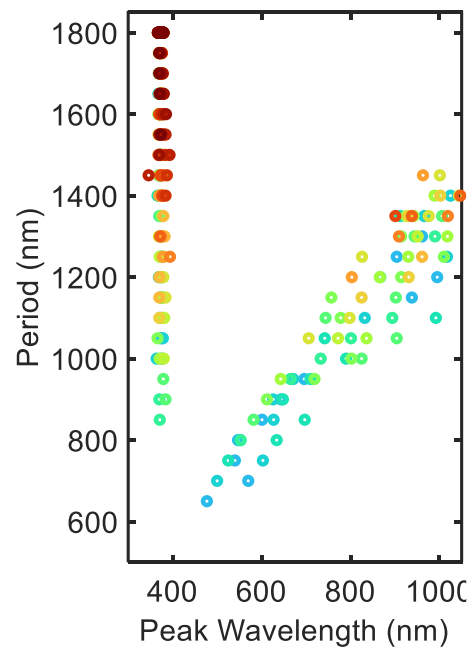**e**

$\lambda$ : 300-1100 nm  
d: **300-1500 nm**  
p: **500:3000 nm**

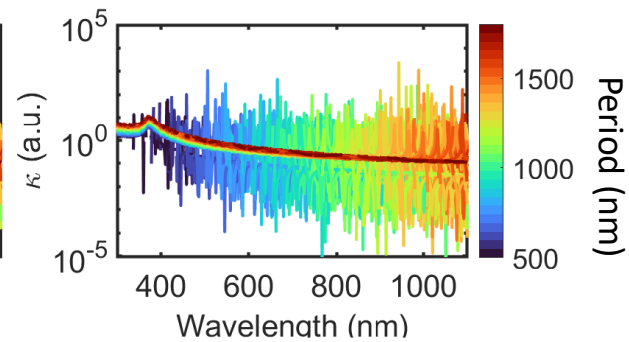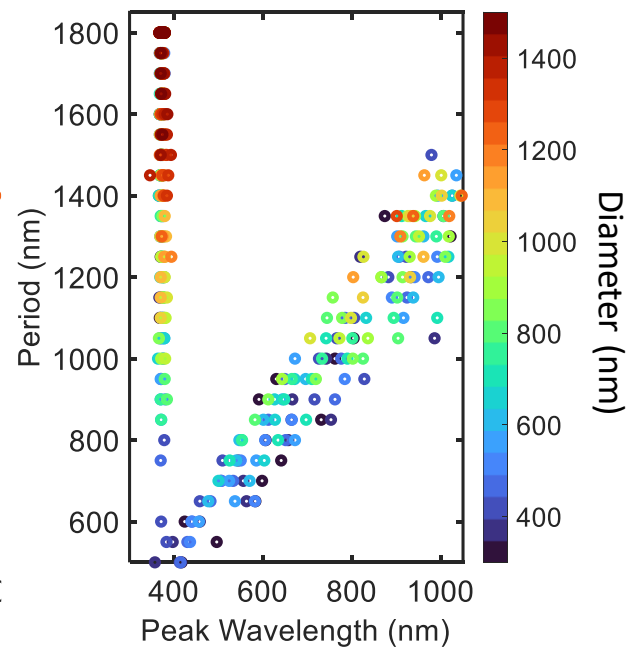

Supplement: Supplementary file 1 — ph4c00453_si_001.zip [file ph4c00453_si_001.zip › Coupling_analysis.pdf]

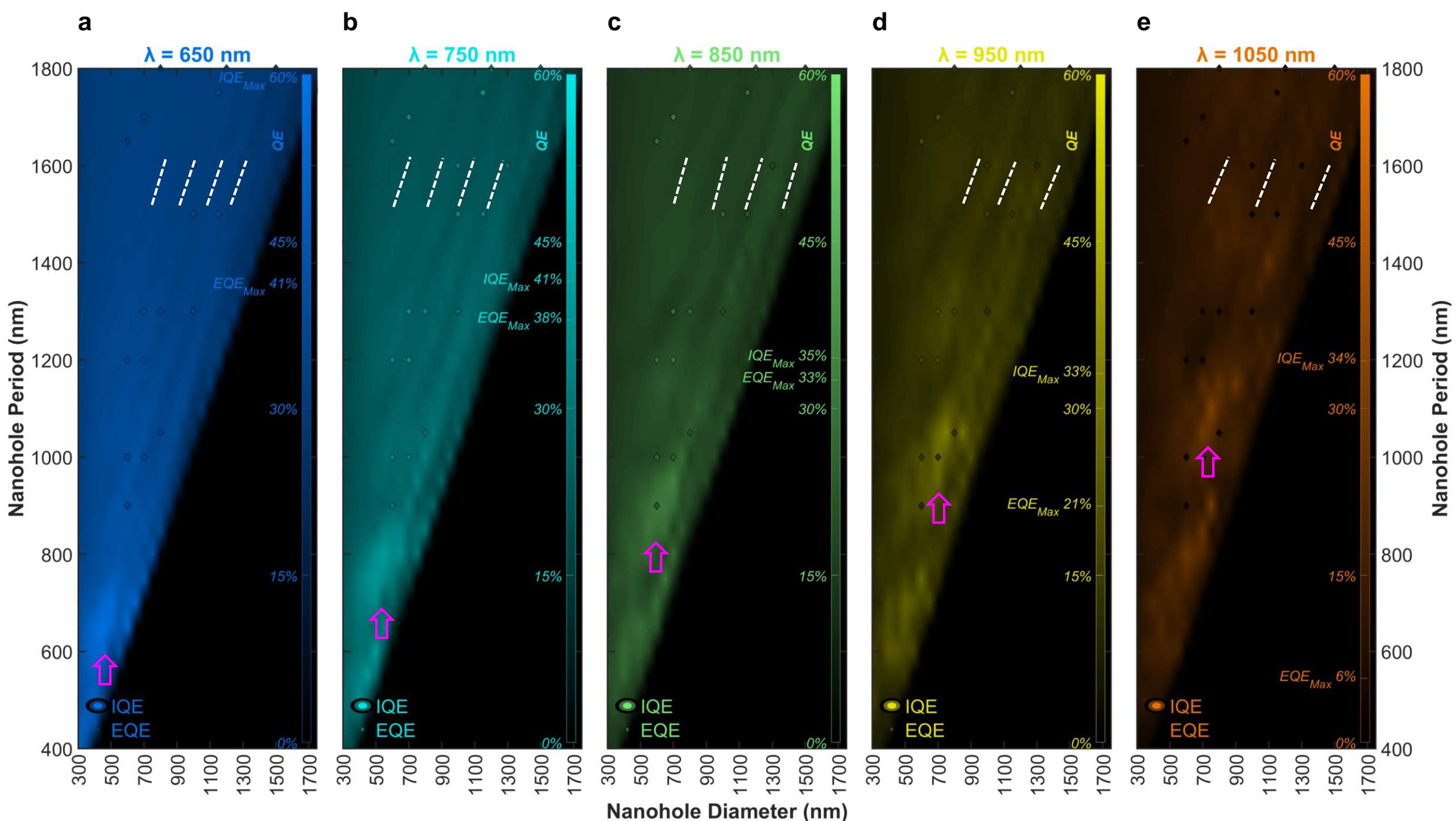

Supplement: Supplementary file 1 — ph4c00453_si_001.zip [file ph4c00453_si_001.zip › EQE_contours.pdf]

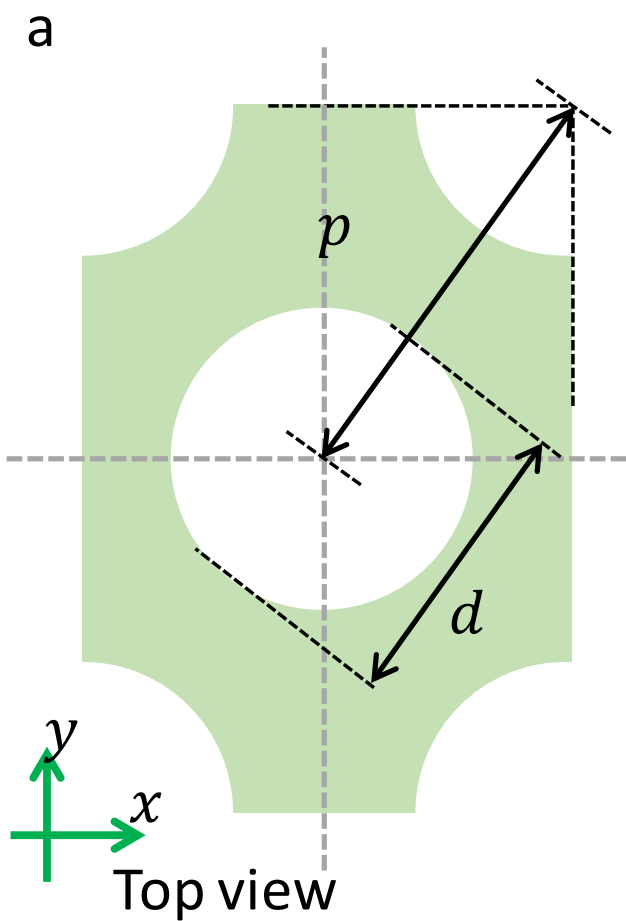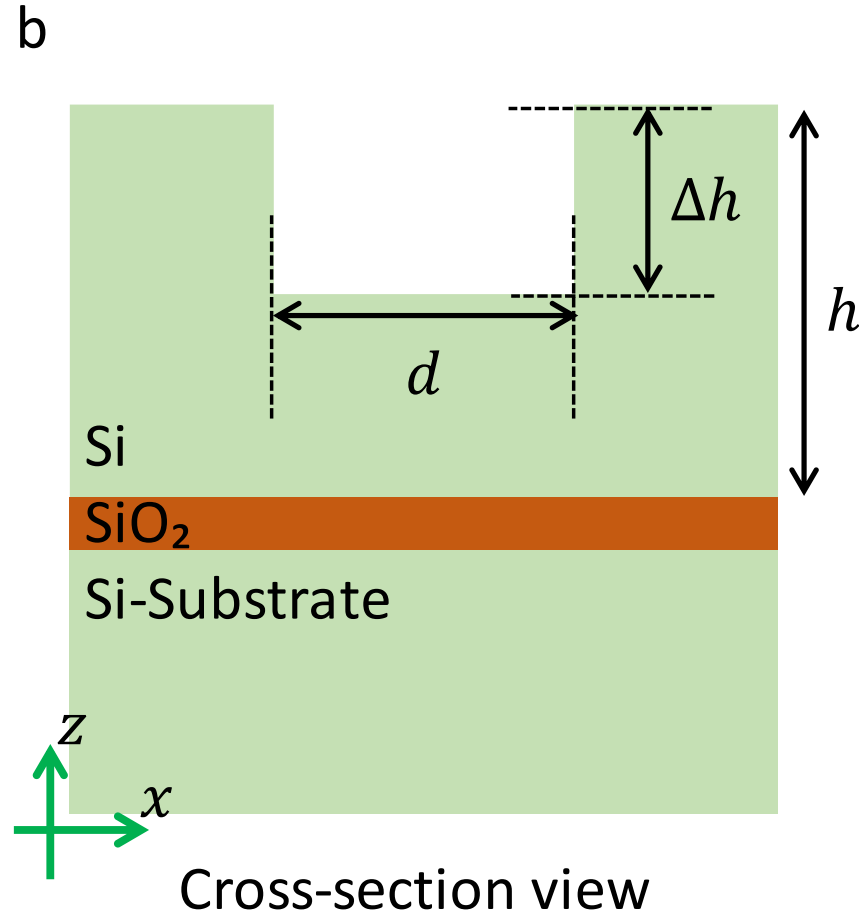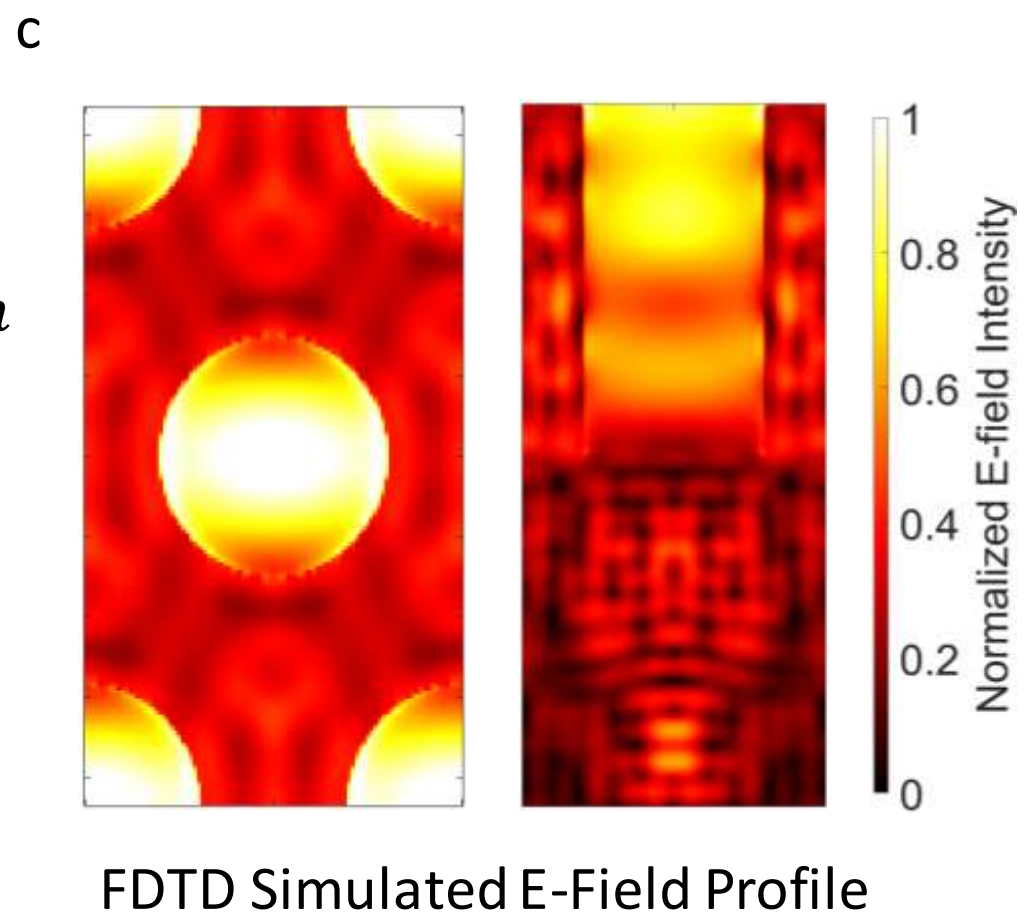

Supplement: Supplementary file 1 — ph4c00453_si_001.zip [file ph4c00453_si_001.zip › FDstructures.pdf]

**a**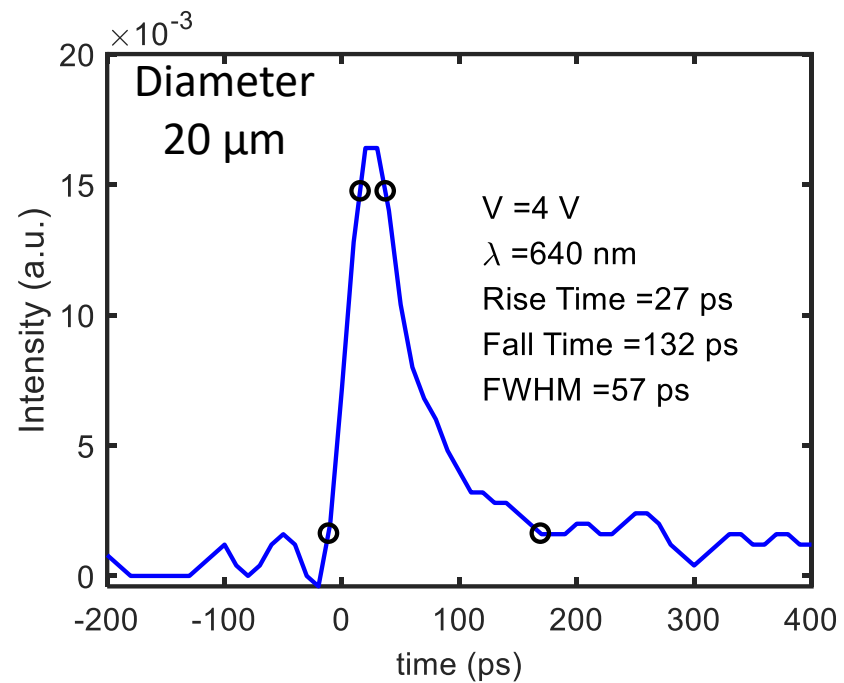**b**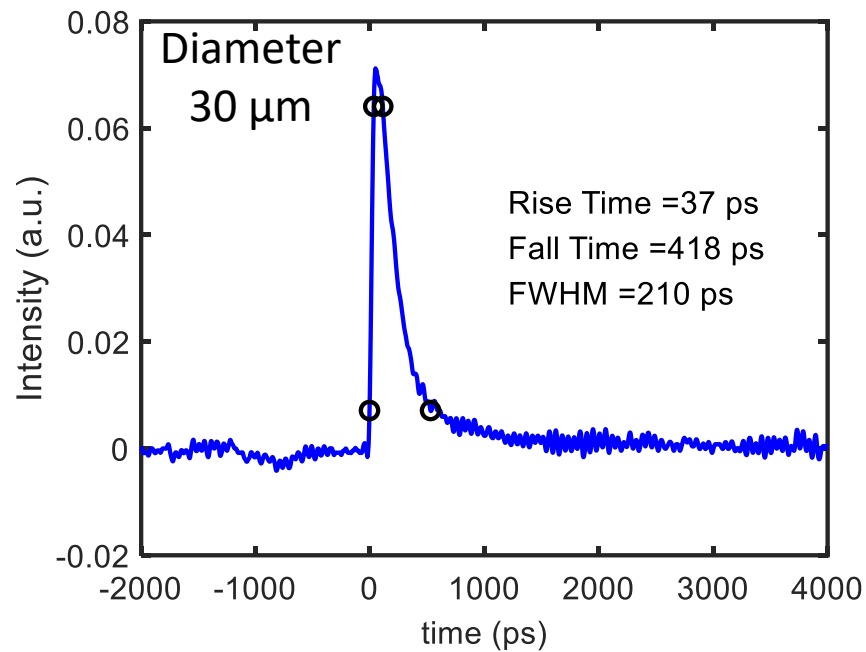**c**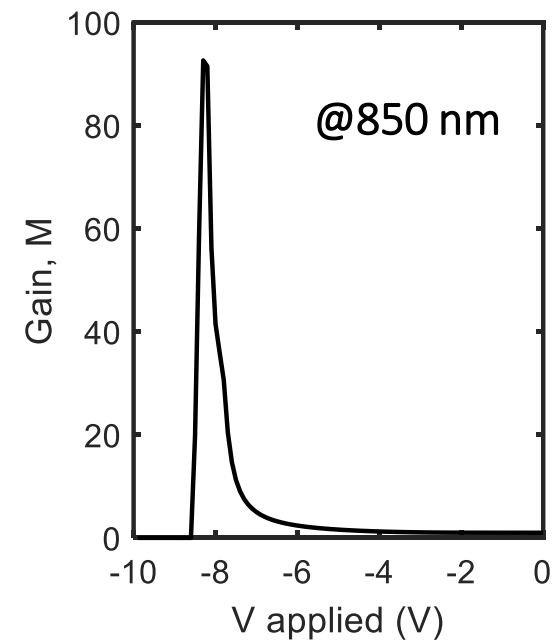

Supplement: Supplementary file 1 — ph4c00453_si_001.zip [file ph4c00453_si_001.zip › high_speed.pdf]

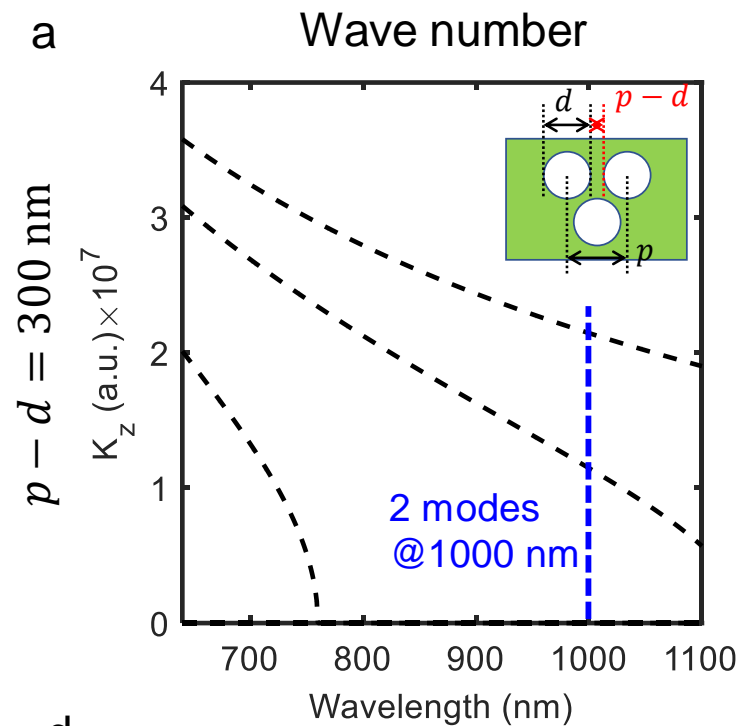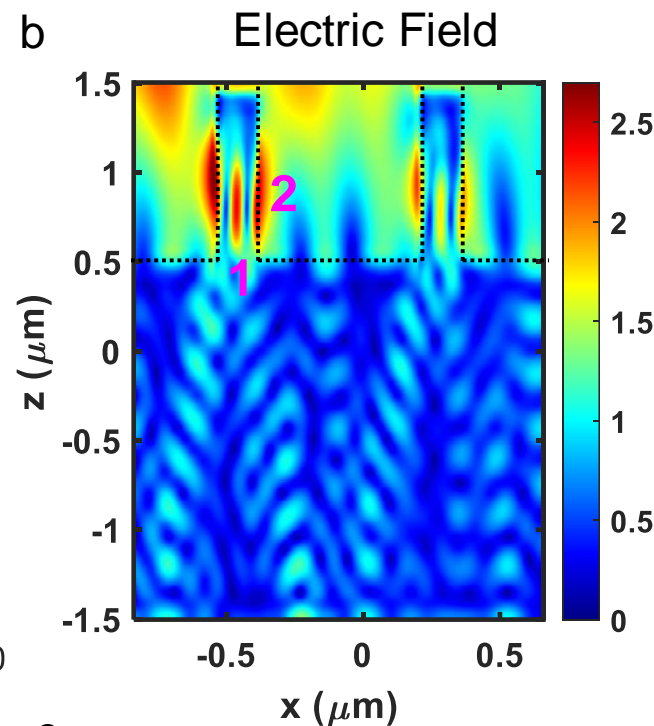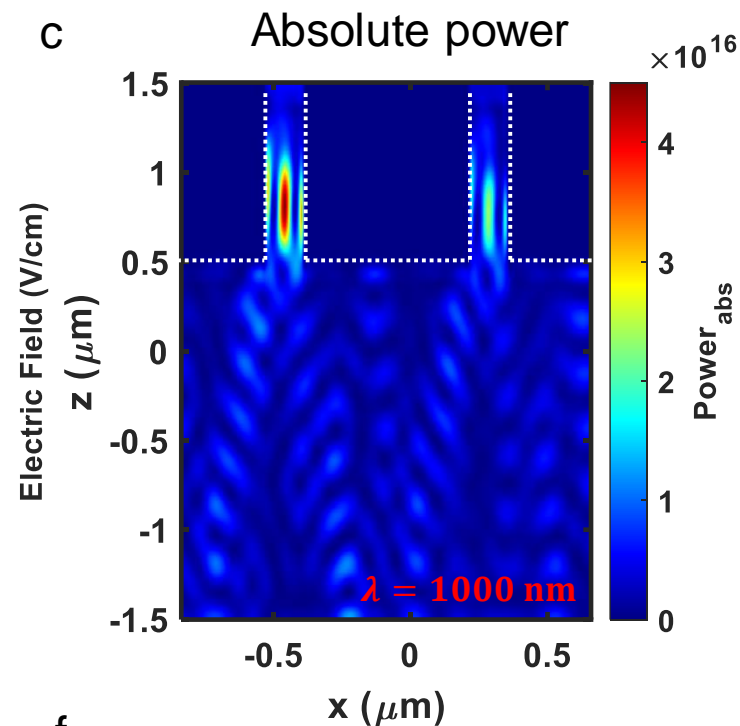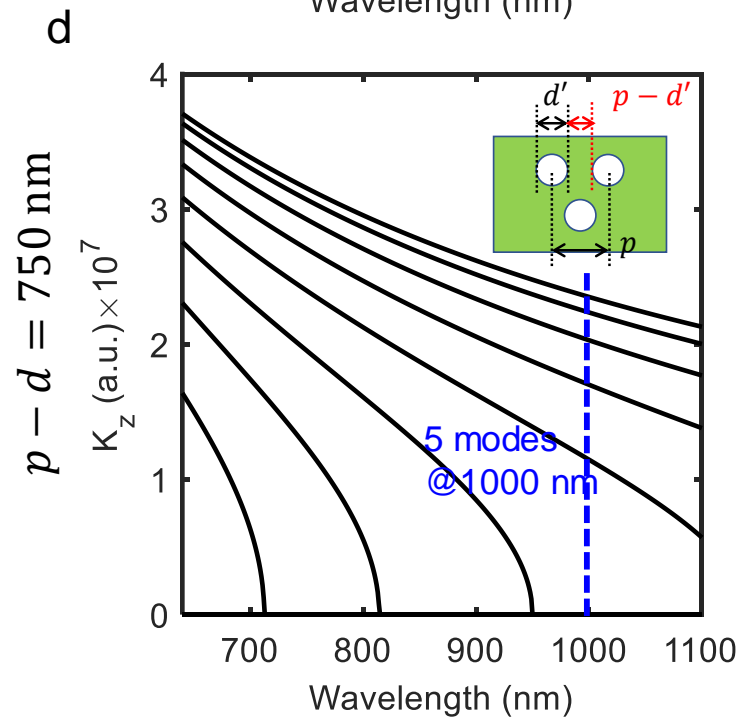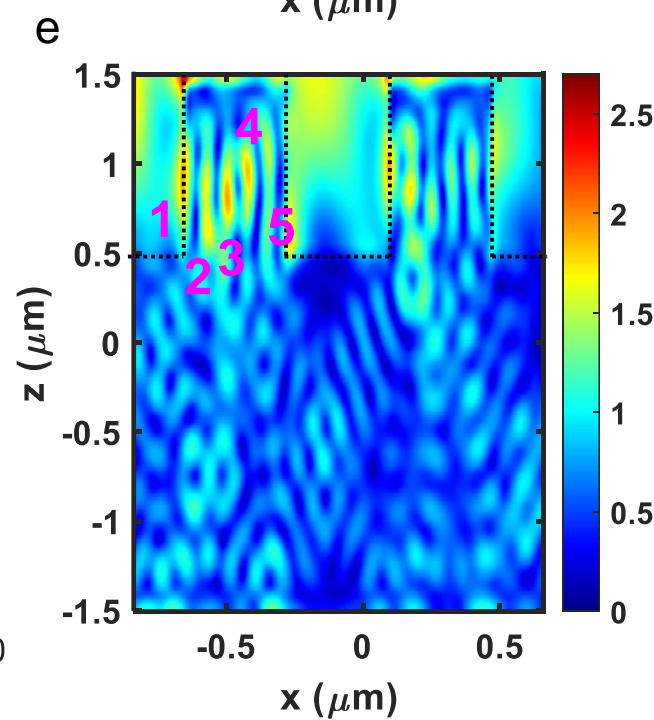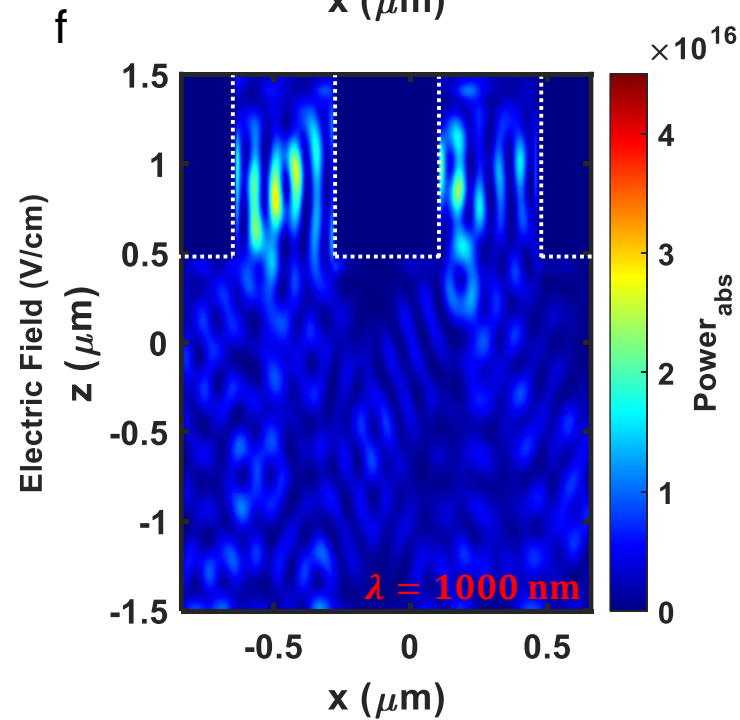

Supplement: Supplementary file 1 — ph4c00453_si_001.zip [file ph4c00453_si_001.zip › pmd_DD.pdf]

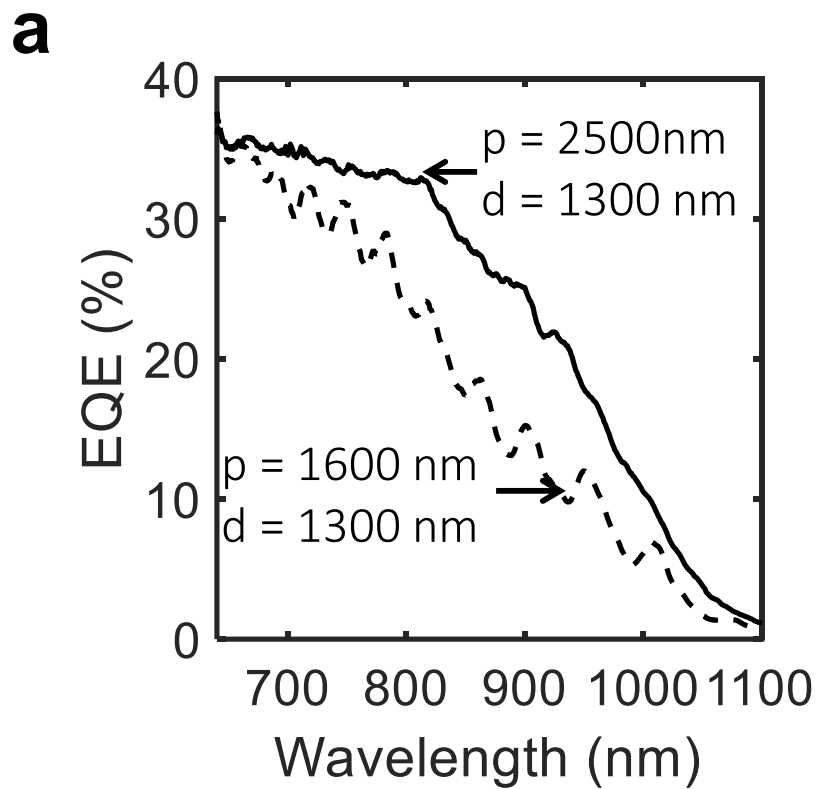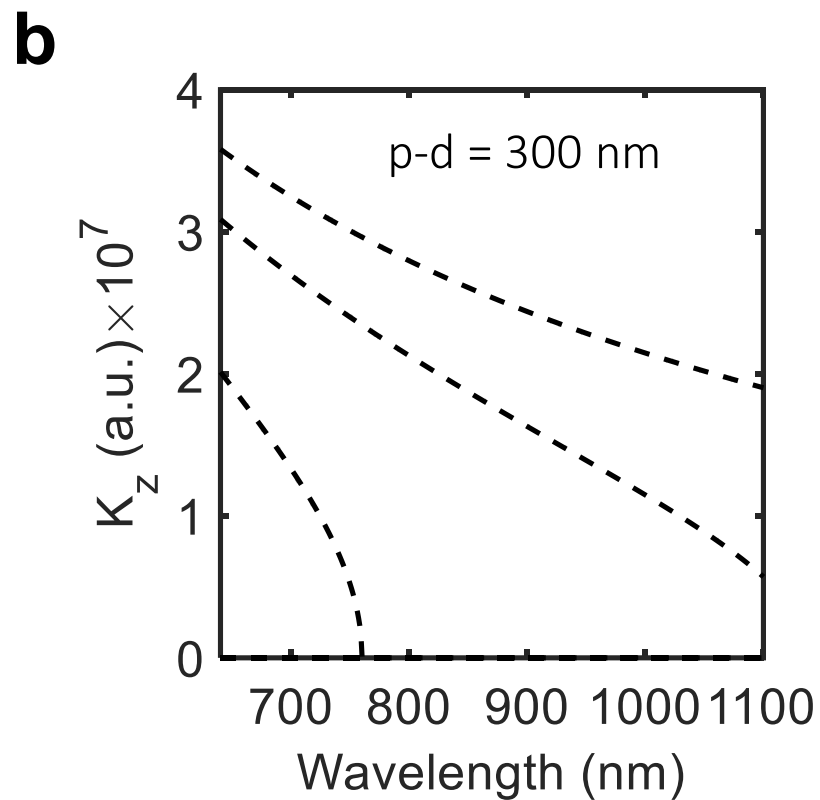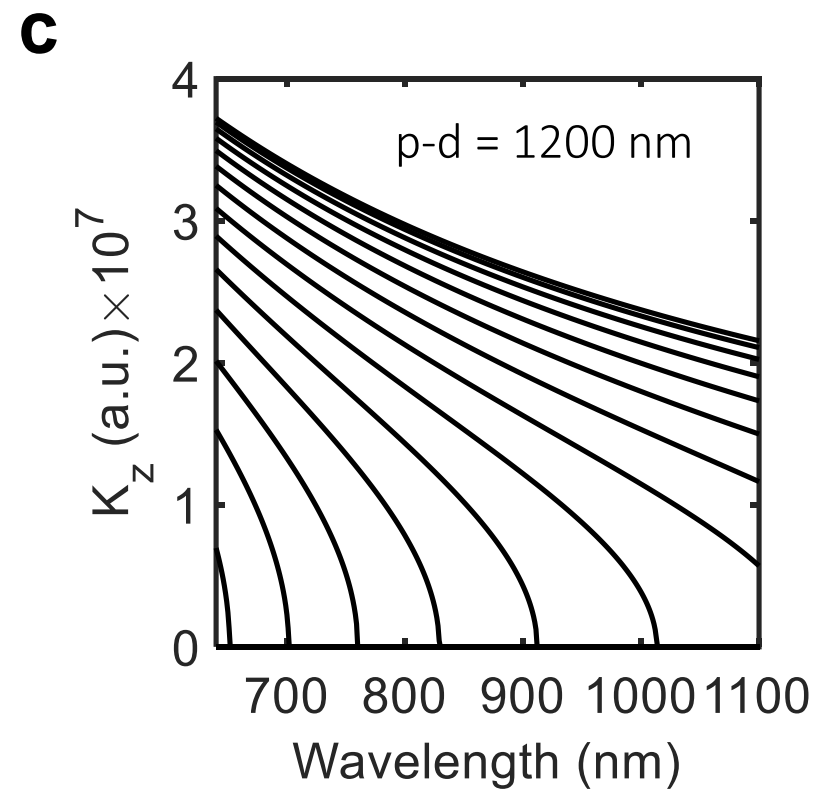

Supplement: Supplementary file 1 — ph4c00453_si_001.zip [file ph4c00453_si_001.zip › pmdexpt.pdf]

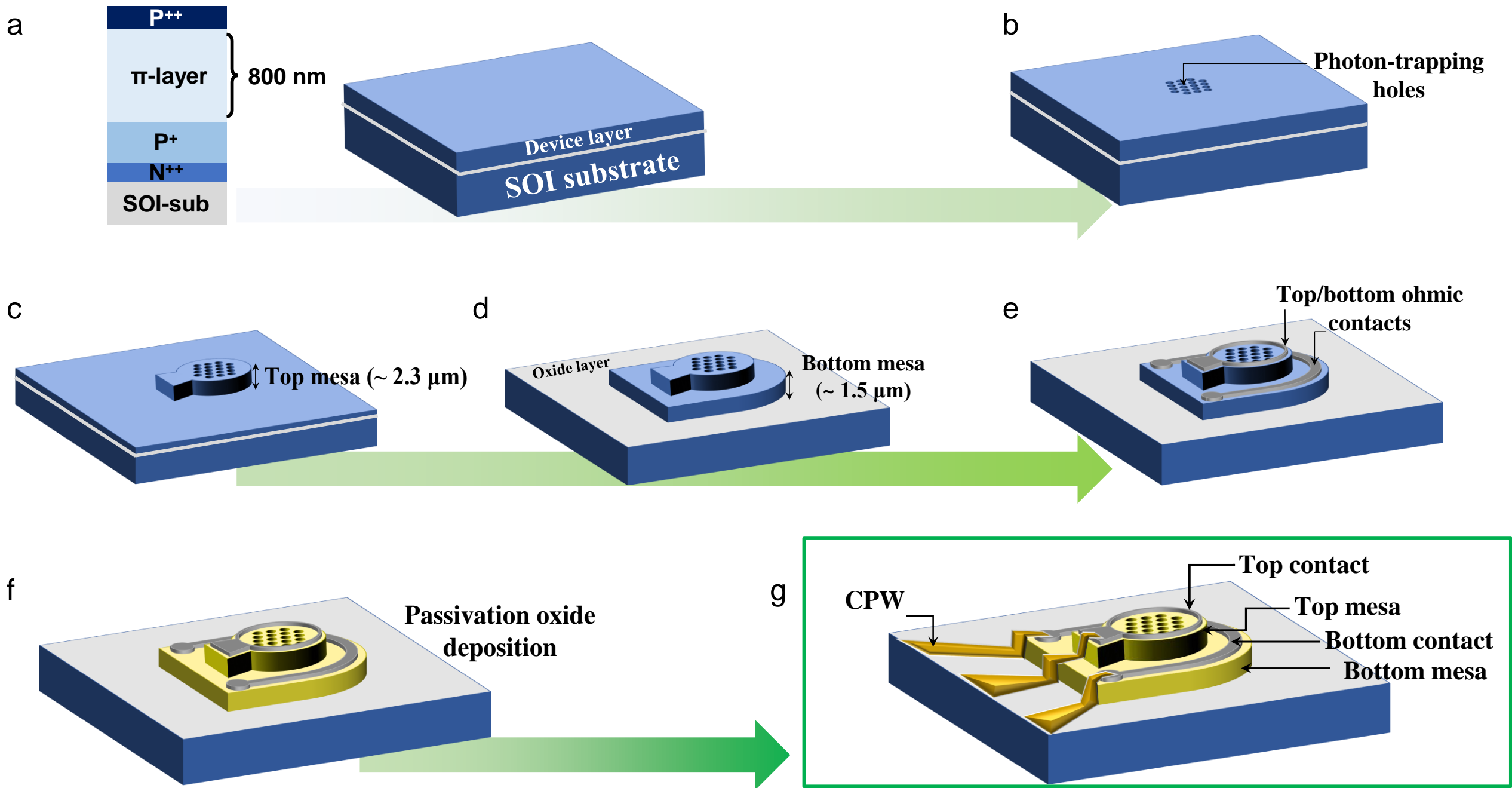

Supplement: Supplementary file 1 — ph4c00453_si_001.zip [file ph4c00453_si_001.zip › Process_flow.pdf]

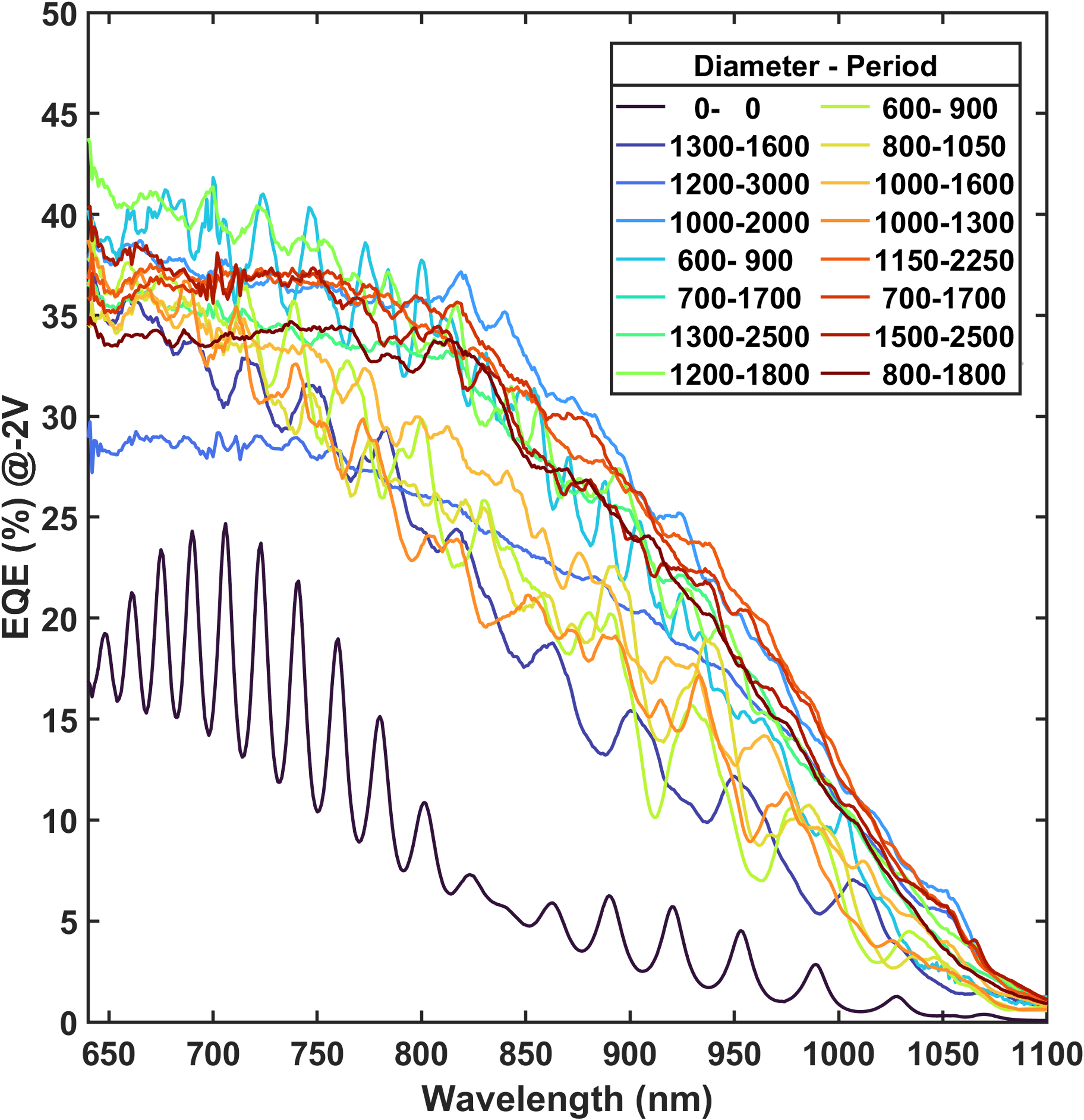

Supplement: Supplementary file 1 — ph4c00453_si_001.zip [file ph4c00453_si_001.zip › EQE.png]

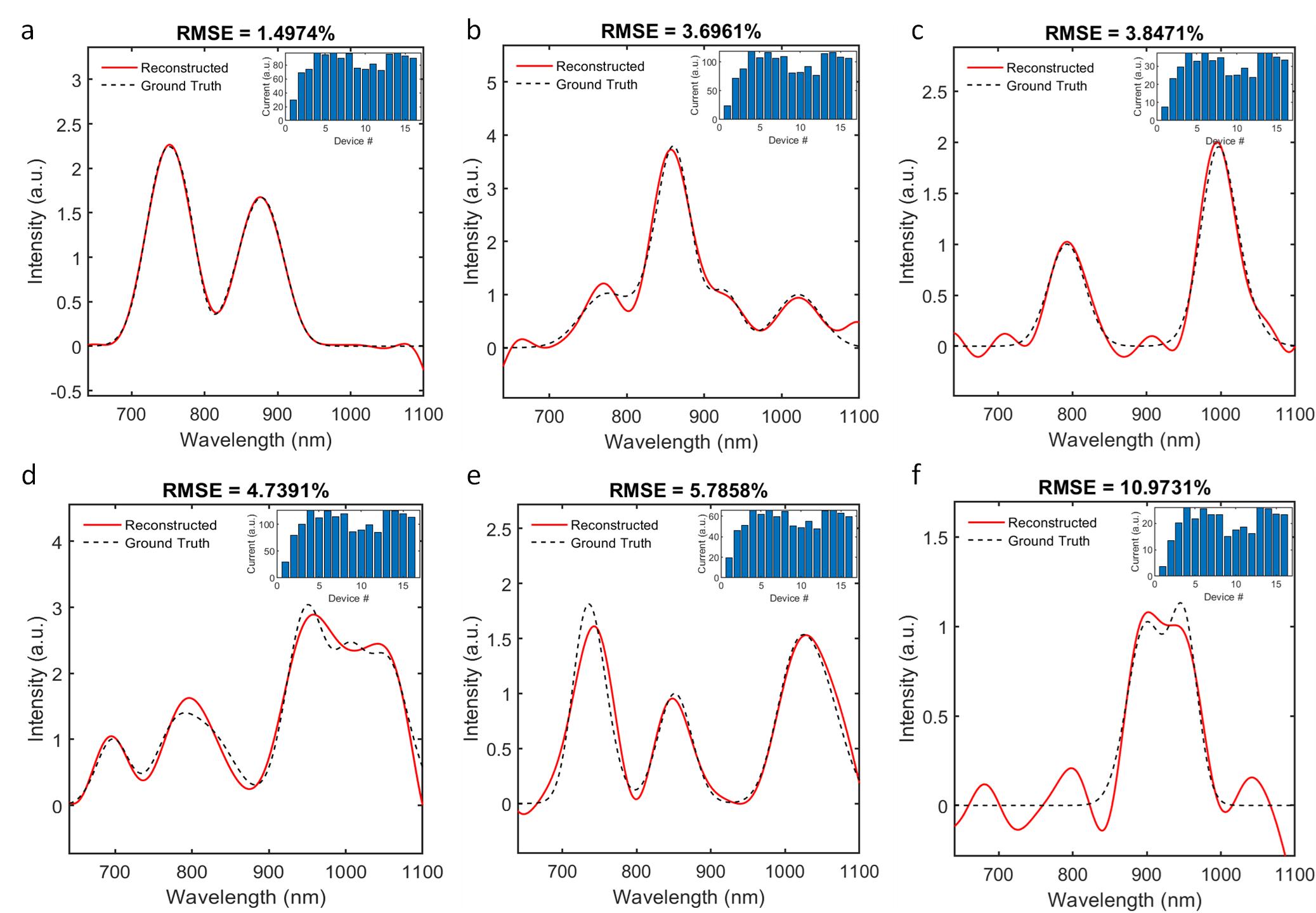

Supplement: Supplementary file 1 — ph4c00453_si_001.zip [file ph4c00453_si_001.zip › Spectral_reconstruction.png]
