# Supplementary material for: Unique Hyperspectral Response Design Enabled by Periodic Surface Textures in Photodiodes
Source: ACS Photonics. 2024 Jun 7;11(6):2497–505. doi: 10.1021/acsphotonics.4c00453 (PMC11191742; doi:10.1021/acsphotonics.4c00453)
Supplement: Supplementary file 2 — ph4c00453_si_002.pdf [file ph4c00453_si_002.pdf]

Unique Hyperspectral Response Design Enabled by Periodic Surface Textures in Photodiodes

Ahasan Ahamed,[†,‡] Amita Rawat,[†,‡] Lisa N. McPhillips,[†] Ahmed S. Mayet,[†] and M. Saif Islam[*,†]

†*Electrical and Computer Engineering, University of California – Davis, Davis, California 95616, USA*

‡*Contributed equally to this work*

E-mail: sislam@ucdavis.edu

Phone: +1 (530) 754-6732. Fax: +1 530-752-8428

Finite-Difference Time-Domain Lumerical simulation details:

The optical simulations were performed in the Lumerical Finite Difference Time Domain (FDTD) module to study the electromagnetic (EM) wave interaction by solving Maxwell's Equation. We have simulated a 3.7 µm thick silicon region placed on 1 µm thick SiO_2 as a buried oxide layer followed by a silicon substrate. We have further introduced the photon-trapping surface textures (PTST) unit-cell into the structure as shown in Fig. S1a-b. We simulated a range of diameter, d (300–1500 nm), and period p (400–2000 nm) of PTST at a fixed depth of 1 µm. We fixed the background refractive index to 1 and simulated 200 fs at 300 K temperature. The absorption data is collected from the intrinsic absorption layer of the device located from 0.2 µm to 1.0 µm depth from the top surface. We used

anti-symmetric, symmetric, and perfectly matched layer (PML) boundary conditions in x, y, and z directions respectively. Figure S1c shows a normalized electric-field intensity profile propagating through the PTST.

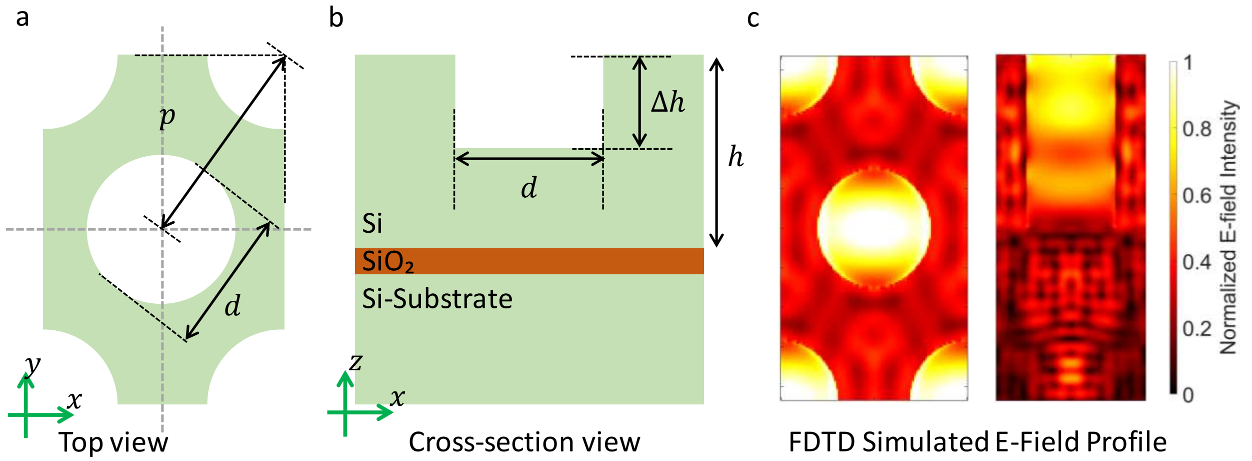

Fig. S1: **PTST-equipped device structure simulated in FDTD**: **a,** Top, and **b,** cross-section view of the device structure simulated showcasing the PTST diameter, d, period, p, hole depth, Δh, and device layers ($Si/SiO_2/Si-subs$); **c,** Normalized electric field propagation profile through the PTST.

Photon-trapping surface texture design and fabrication

We design the PTST lattice for the devices based on the FDTD simulation results. We have chosen around 40 different PTST diameter-period combinations for the mask design and fabricated them. Due to fabrication limitations, we are limited to a minimum of 600 nm hole diameter and 900 nm hole period. A minimum of 300 nm separation was required to maintain the good health of the nanoholes and prevent them from merging with the nearby holes. We also include several designs to include sparse hole densities and extend the hole period up to 3000 nm.

In Fig. S2, we have plotted the EM wave absorption efficiency simulated in the FDTD framework for a range of PTST diameters, d, and periods, p for wavelengths ranging from 650 nm to 1050 nm. The recurring dark and bright contours in the EQE show the unique wavelength selectivity enabled by PTST dimensions and photonic bandgap formation (marked by white dashed lines). The shift in the peak absorption efficiency position in p and d space (marked by the magenta arrows) shows a need for a larger PTST period with increased illumination wavelength.

We designed the device stack as shown in Fig. S3a with a thin absorber layer (thickness

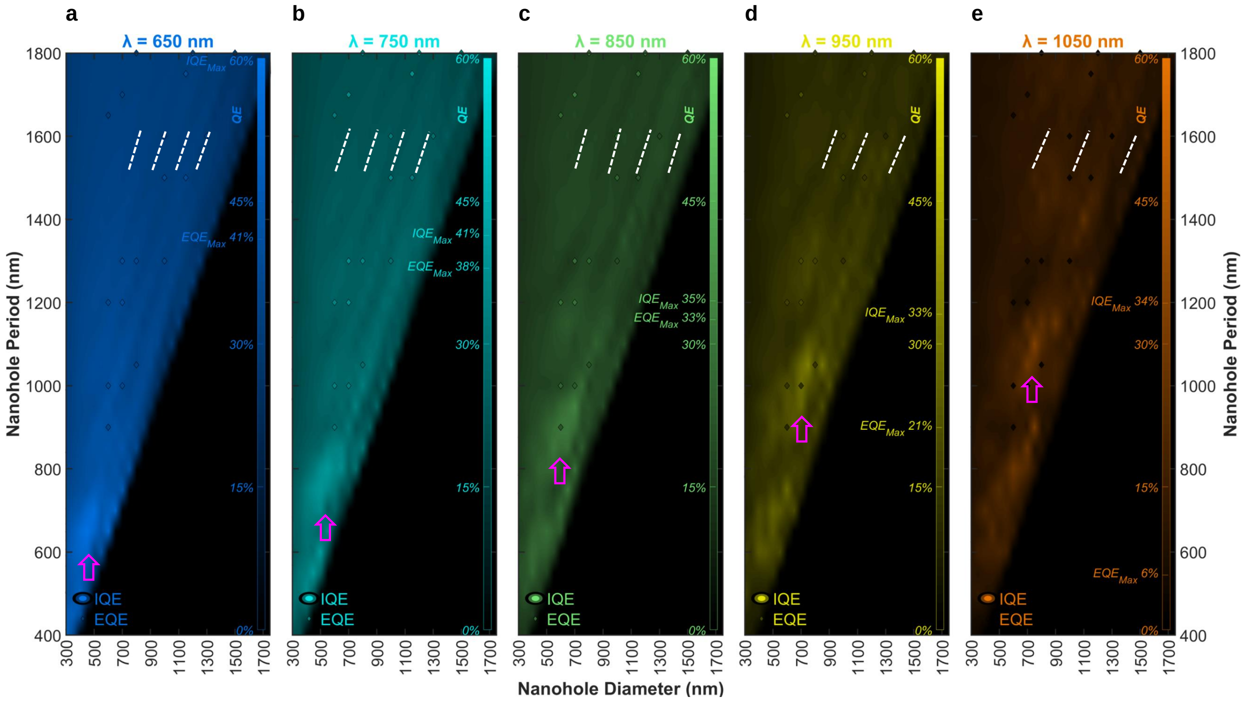

Fig. S2: **Optical absorption efficiency**: **a-e,** Optical absorption obtained from FDTD simulation of the device with nanoholes varied in periodicity from 400 nm to 1800 nm and diameter from 300 nm to 1750 nm. The absorption efficiency is shown for **a,** 650 nm, **b,** 750 nm, **c,** 850 nm, **d,** 950 nm, and **e,** 1050 nm illumination wavelengths respectively. The diamond markers indicate the EQE obtained from fabricated devices with a defined period and diameter of micro/nano-holes. The dark region originating from the bottom right in each plot represents unfeasible PTST cases where $d > p$. The white dashed lines highlight the effect of photonic bandgap formation where a certain wavelength does not get absorbed efficiently due to PTST. Further, the rising magenta arrow shows the position of the peak efficiency period increasing with the increased illumination wavelength.

∼800 nm) to fabricate a high-speed photodiode. We used Deep Ultraviolet (DUV) Stepper

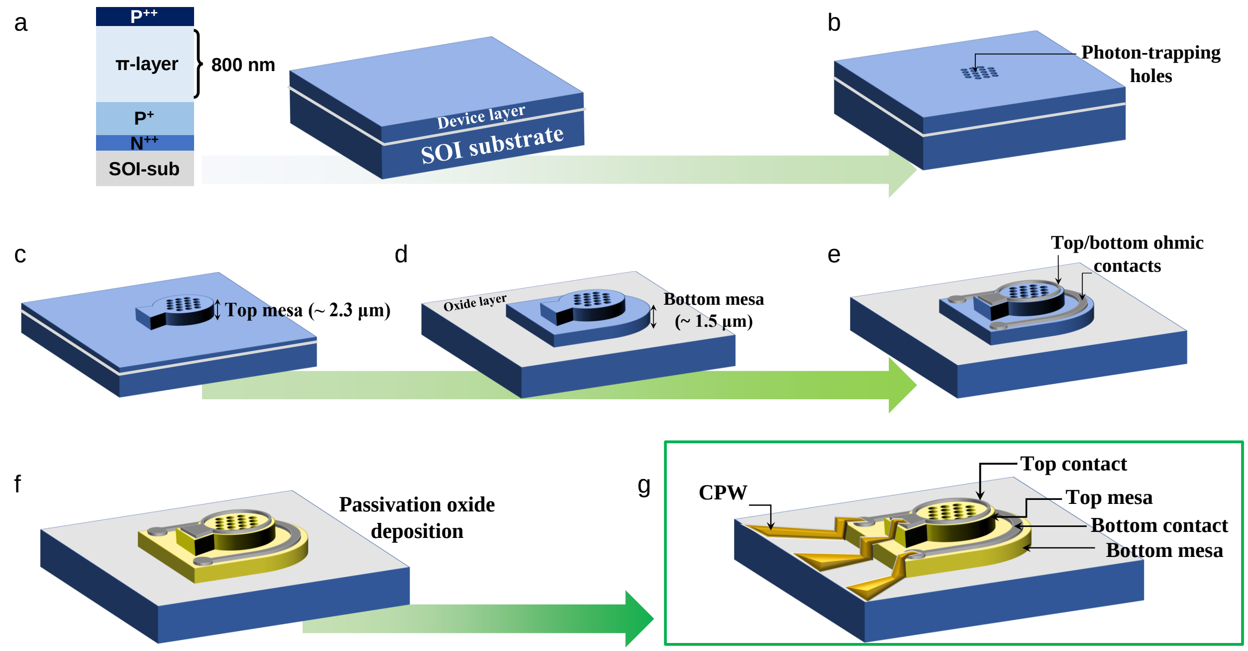

Fig. S3: **Fabrication process flow**: Step-wise photodiode fabrication process flow designed to ensure compatibility with complementary metal oxide semiconductor process-line. **a,** The doping arrangement highlighting the absorber layer thickness in the photodiodes and the silicon-on-insulator wafer used to isolate the active device region from the bulk-Si substrate for preventing substrate leakage; **b,** the photon-trapping surface texture (PTST) array is patterned using non-contact projection mode ultraviolet lithography process; **c,** the top mesa is patterned in alignment with the PTST array and etched in order to reveal bottom highly doped layer using inductively coupled reactive ion etching (ICPRIE) process; **d,** in alignment with the top mesa, the bottom mesa is patterned and etched to isolate devices from one-another; **e,** after the mesa patterning, we patterned both top and the bottom metal contact in alignment with the mesas, deposited aluminum using e-beam evaporation process (the work function of Al (4.15 eV) lies close to the midgap of silicon and results in ohmic contact both for p and n type dopings) and formed the contacts using the lift-off process. **f,** The photodiode fabrication process insofar involves an intensive dry etching process that can give rise to surface states and result in high leakage current, therefore, to passivate the surface states due to the dangling bonds, we conformally coated SiO_2 layer in a chemical vapor deposition chamber; **g,** finally, we patterned the co-planar waveguide (CPW) contacts, selectively etch the SiO_2 from the top of both the metal contact and formed Al-CPW contacts. Our previous reference[1] shows further detail of the fabrication process.

system to pattern cylindrical PT nanoholes and etched the holes (hole depth $\sim$1.0 µm) using an inductively coupled plasma-reactive ion etching (ICP-RIE) process. In alignment with the PTST, we patterned and etch the top mesa to expose the bottom highly doped region.

Next, we patterned and etch the bottom mesa in alignment with the top mesa to isolate one device from the other. Further, we pattern and form the top and bottom contact using a standard metal deposition and the lift-off processes. To passivate the damage caused by multiple intensive dry etching processes we perform SiO_2 based surface passivation in a chemical vapor deposition system. Finally, we pattern the co-planar waveguide contacts for high-speed measurements. Figure S3 describes the process steps used. Further details are presented in our previous work.[1]

Photonic crystals, diffractive optics, and meta-optics are all terms used to describe different aspects of light manipulation. Photonic crystals refer to 2D and 3D structures where concepts like reciprocal space, Brillouin zone, dispersion relations, Bloch wavefunctions, Van Hove singularities, etc. are applicable.[2] In our study, we have not investigated the PTST thoroughly to be able to categorize it as photonic crystals. Diffractive optics is a broader term that includes photonic crystals and other surface structures such as gratings and surface plasmons.[3] While this surface structure falls under this category, PTST is preferred for its functional specificity. Meta-optics, are sub-wavelength surface structures used in beam deflection, focusing, wavefront engineering, phase control, etc.[4] These PTSTs on the other hand, are used to deflect electromagnetic waves laterally for enhanced absorption to generate unique absorption trends, a key aspect essential in hyperspectral imaging.

Device performance evaluation

The transient response of the fabricated PTST-equipped photodiodes is plotted in Fig. S4a,b. We have achieved a response time as low as 27 ps, and a full-width half maxima (FWHM) of 57 ps. The fall time of the device is reported to be 132 ps, due to prolonged hole-recombination time. The bandwidth of the device is estimated to be ~2.7 GHz (Bandwidth $= 2.2/(2\times\pi\times$Fall-time)). The multiplication gain, M, obtained from the device at a fixed

illumination wavelength of 850 nm is ∼90 as shown in Fig. S4c. The high speed and high multiplication gain along with fine spectral resolution make these devices a suitable candidate for hyper/multispectral imaging applications.

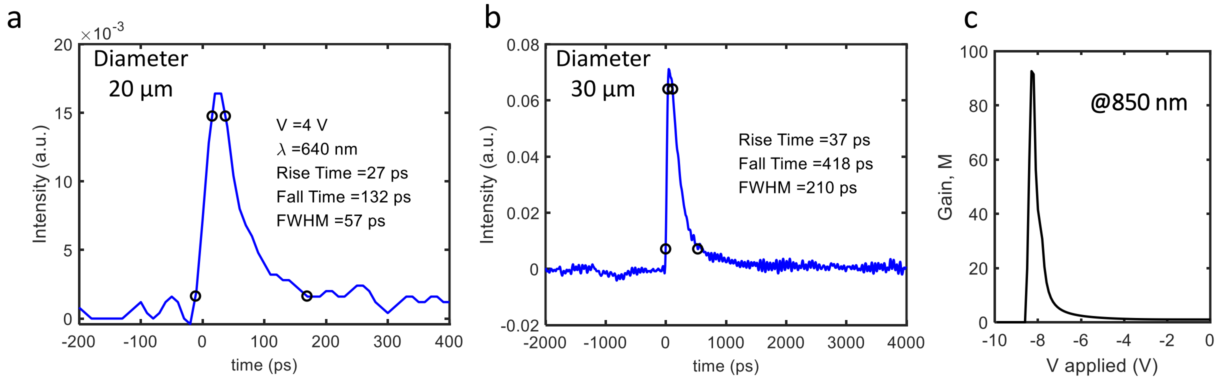

Fig. S4: **Transient response:** The transient response time of **a,** 20 µm and **b,** 30 µm are shown by illuminating the device with 640 nm wavelength pulses. **c,** Multiplication gain obtained for 850 nm wavelength illumination.

Fabry-Pérot Modulation Impact on EQE

In Fig. S5a-d, we show a gradual dissolution of fringes present in the external quantum efficiency (EQE) profile of the flat device with the introduction of PTST and increasing the PTST density (decreasing the PTST periodicity from 3000 nm to 2000 nm, while fixing the PTST diameter to 1000 nm). Introducing the PTST causes a perturbation in the Fabry–Pérot cavity facilitated by the Si/SiO_2 interface in the flat photodiode that results in unique EQE profiles. The PTST density governs the extent of perturbation and the extent of dissolution of Fabry–Pérot cavity resonance.

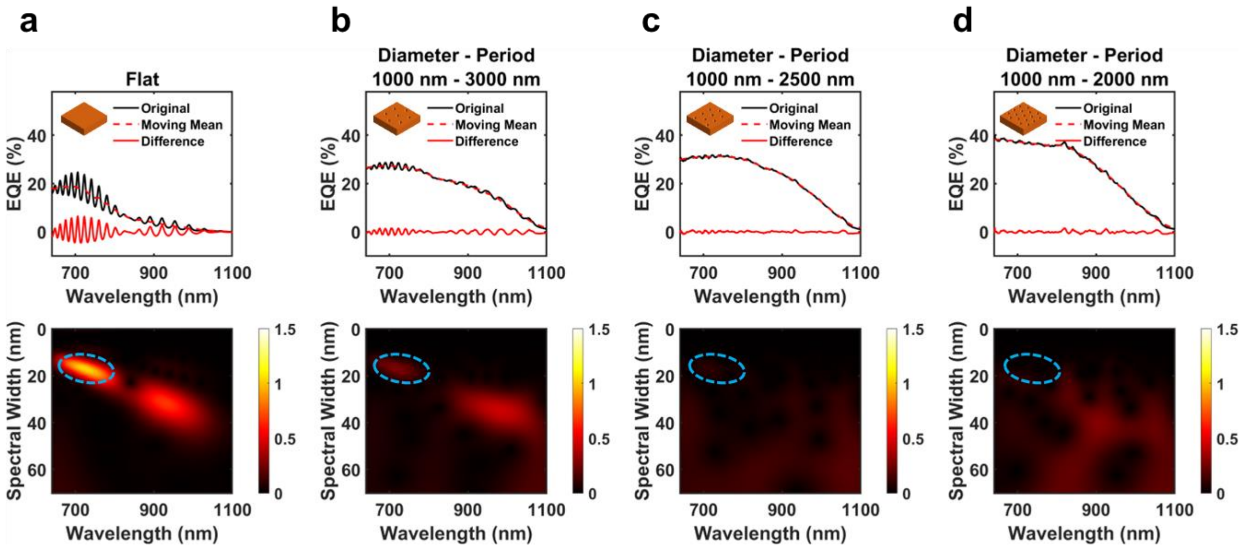

Fig. S5: **Fabry–Pérot Effect Modulation:** The EQE (top) and respective Morlet wavelet transform (bottom) are shown for, **a,** A flat, and for photodiodes with PTST by fixing the diameter to 1000 nm and varying periods such as **b,** 3000 nm, **c,** 2500 nm, and **d,** 2000 nm. The EQE (black) and ΔEQE (red) curves show prominent fringes due to the vertical resonance triggered by the Fabry-Pérot effect in the flat device. In contrast, the fringes diminish as PTST density increases with reduced periodicity from left to right. The small modulation in the EQE spectra has been transformed into evidential uniqueness using the Morlet wavelet transform. The predictable vertical resonance in the flat device has been transformed into a unique EQE spectral response by introducing the PTST features.

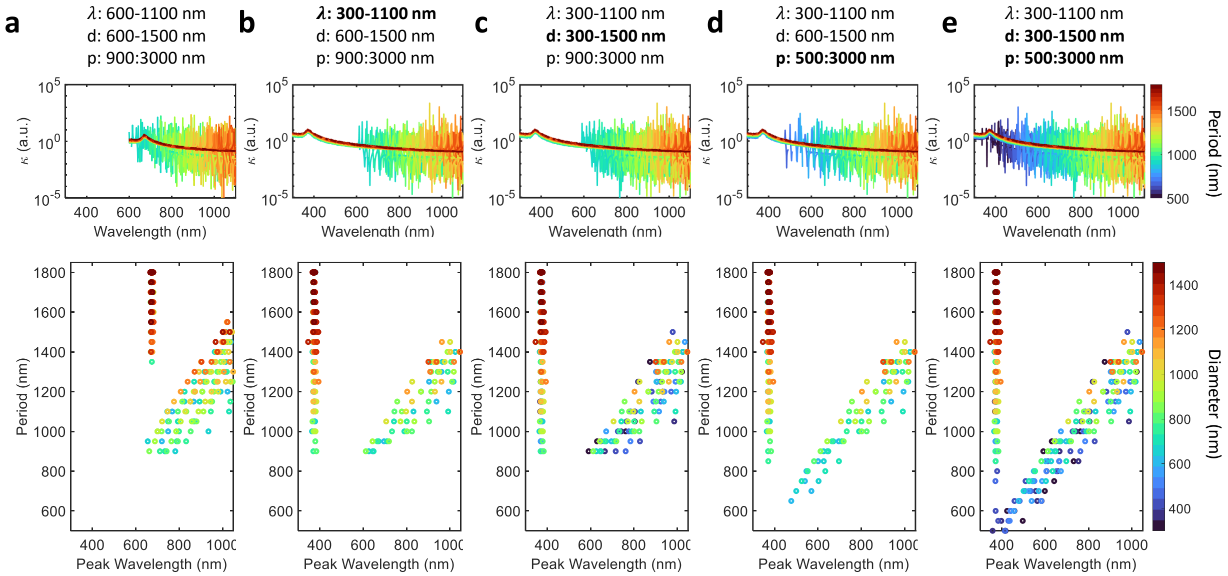

Fig. S6: **Coupling analysis:** We present a detailed behavioral analysis of the coupling coefficient modulation with illumination wavelength for a range of PTST diameters, d, and periods, p. **a,** coupling coefficient (top) and PTST period versus peak wavelength trend (bottom) for a limited set of PTST dimensions (d: 600-1500 nm; p: 900-3000 nm; λ: 600-1100 nm). We observe two coupling scenarios. The first is a weakly coupled scenario that results in maximum absorption at the lowest illumination wavelength, and the coupling coefficient follows a monotonous trend (consistent with the intrinsic absorption coefficient of the material). The second one is a strongly coupled scenario where the peak absorption wavelength shows a linear trend with the PTST period, and the coupling coefficient shows a selective enhancement per wavelength. **b,** Changing the limits of illumination wavelength to 300-1100 nm while keeping the d and the p same as **(a)**, shifts the peak of weakly coupled wavelength to ∼375 nm. **c,** Next, changing the range of d to 300-1100 nm slightly shifts the strongly coupled wavelengths toward the left (top). **d,** Further, changing the range of p to 500-3000 nm shows a noticeable left shift enabling strong coupling in the lower wavelengths (top). **e,** Finally, changing p, d, and λ ranges results in continuous wavelength coupling throughout the spectrum. The wavelength coupling coefficient, κ is a strong function of the PTST period, p and the peak wavelength is a linear function of p when strongly coupled. The κ is a weak function of PTST diameter, d.

Coupling coefficient modulation

In Fig. S6a, we show the analytically calculated coupling coefficient, κ for a range of λ, d, and p. In Fig. S6a, we limit the range of λ, d, and p to 600-1100 nm, 600-1500 nm, and 900-3000 nm respectively. We observe the κ resulting from the weak coupling shows a monotonous trend as opposed to the discrete spikes in the κ trend resulting from the strong coupling (Fig. S6a,(top)). The peak coupling wavelength for a given PTST (at fixed d and p), when strongly coupled, shows a linear relationship with the period, p, whereas, the peak coupling wavelength becomes the lowest illumination wavelength (peak wavelength = 600 nm in Fig. S6a) in the case of weak coupling. This selective nature of coupling results in two slopes in the period versus peak wavelength trend as shown in Fig. S6a,(bottom). Next, we changed the range of λ to 300-1100 nm as shown in Fig. S6b. Modifying the lower limit of illumination wavelength only changes the peak wavelengths resulting from a weak coupling trend as shown in Fig. S6b,(top). Whereas, the strongly coupled wavelengths are constrained by the lower limits of d and p. The updated weakly coupled wavelengths as a function of the period have been shifted close to the lower limit, i.e., $\sim$375 nm as plotted in Fig. S6b,(bottom). The impact of this shift in weakly coupled peak wavelengths in the period vs. peak wavelength trend repopulates the strongly coupled wavelengths as well. Further, we changed the lower limit of the d to 300 nm (i.e., range of d: 300-1500 nm), and observed a slight left shift in the strongly coupled scenario that qualifies shorter wavelengths to exhibit strong coupling as shown in Fig. S6c. The weakly coupled scenarios remain consistent, as the limits of the λ do not change, and strongly coupled scenarios remain linear with a proportionate population at shorter peak wavelengths. Furthermore, we reduce the lower limit of p to 500 nm, while restoring the lower limit of d to 600 nm. Due to these unusual limits, some PTST arrangements will not be feasible (as $p<d$). We observe a notable left shift in the coupling coefficient trend for the feasible PTST arrangements enabling even shorter wavelength to exhibit coupling as shown in Fig. S6d,(top). The impact of this prominent left shift in the strongly coupled scenario is reflected in the period vs. peak wavelength trend. This shows

that the EM wave coupling is a strong function of the PTST period, p, and a weak function of the PTST diameter, d. Finally, we reduced the lower limits of both d and p to show a strong coupling throughout the wavelength range in Fig. S6e,(top), and the period vs. peak wavelength trend for strongly coupled cases has been stretched left as shown in Fig. S6e,(bottom). With this, we conclude that the coupling coefficient of EM wave for a device with a given PTST arrangement exhibits both weak and strong couplings. The weak coupling is independent of the PTST dimensions (i.e., d and p) and only depends on the range of illumination wavelength and intrinsic absorption coefficient of the material. Whereas, the strong coupling favors certain wavelengths, and the peak coupling wavelength shows a linear relationship with the PTST period, p.

PTST selection scheme for hyperspectral imaging

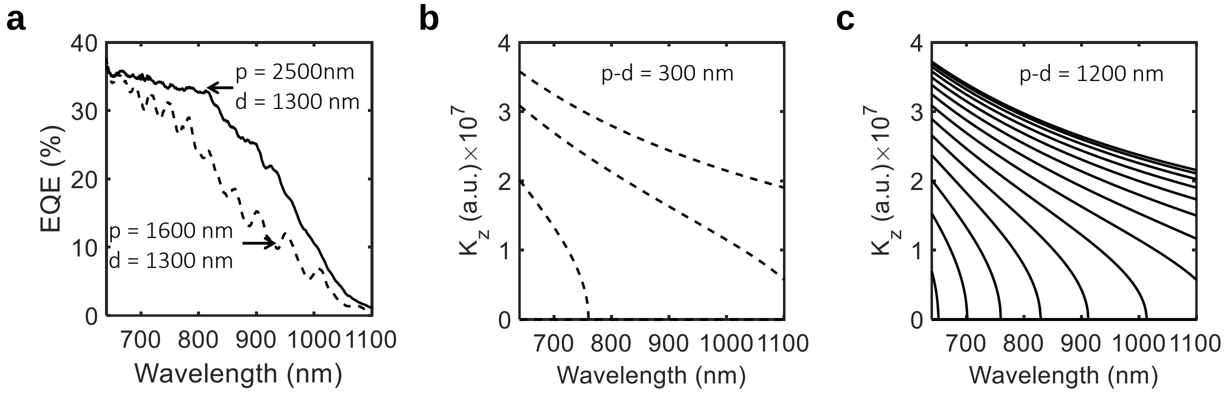

Fig. S7: **Manipulation of EQE Fringes through Control of TE Mode Propagation**: We have investigated the influence of the $p-d$ in various PTST configurations on the fringes of EQE profiles. **a,** Experimentally extracted EQE profile for two photodiodes with two different $p-d$ configurations, 1) $p-d = 300$ nm; 2) $p-d = 1200$ nm. The wider the $p-d$, the more the number of allowed TE modes in the $p-d$ region. The dispersion relationship for a range of illumination wavelengths for **b,** $p-d = 300$ nm and **c,** $p-d = 1200$ nm. The dispersion relationship shows that a 1200 nm wide $p-d$ region will accommodate a larger number of TE modes as against the narrow $p-d$ region of 300 nm.

The fundamental requirement in hyperspectral imaging is to design a photodetector system capable of decerning and detecting a wide range of emission wavelengths from the subject.[5–13] To address this need, we introduce the PTST design method, a novel approach aimed at customizing the spectral response of specific photodiodes. By harnessing their unique responses, we can reconstruct the spectra of the incident illumination wavelengths. The PTST selection and photodiode placement scheme are outlined below.

- The selection of PTST dimensions is crucial to match the required emission wavelength range. For instance, to facilitate spectroscopy for longer wavelengths, such as 1050 nm, the PTST diameter should be approximately 1000-1200 nm, and the periodicity should be around 1400-1600 nm, as illustrated in Figure S6.

- When determining the diameter and periodicity of the PTST, it is crucial to maintain a moderate width for the critical $p-d$ region, as depicted in Figure S7. An excessively wide $p-d$ region would permit a larger number of TE modes, thereby diminishing the fringes in the EQE profile and reducing the device-to-device uniqueness. Conversely, a narrower $p-d$ selection would result in higher fringes, potentially leading to the loss of uniqueness among different devices.

- The number of unique photodiodes necessary for predicting a specific wavelength is inversely proportional to the minimal wavelength resolution. Consequently, there should be an upper limit on the number of photodiodes, which must be determined based on the trade-off between wavelength resolution and the designated chip area for the detector array.

- The arrangement of the photodiode array must ensure uniform illumination for each photodiode.

Spectral reconstruction scheme

To reconstruct the spectrum, we have used a 4×4 unique photodiode array (This is to be noted that we have not fabricated the array of 4×4 detectors. Instead, we have used 16 individual detectors fabricated on a common substrate.). The absorption spectrum in the form of EQE for individual detectors is presented in Fig. S8. A linear approximation method has been employed to reconstruct the spectrum using the photocurrent data from these 16 unique photodiodes.[14,15]

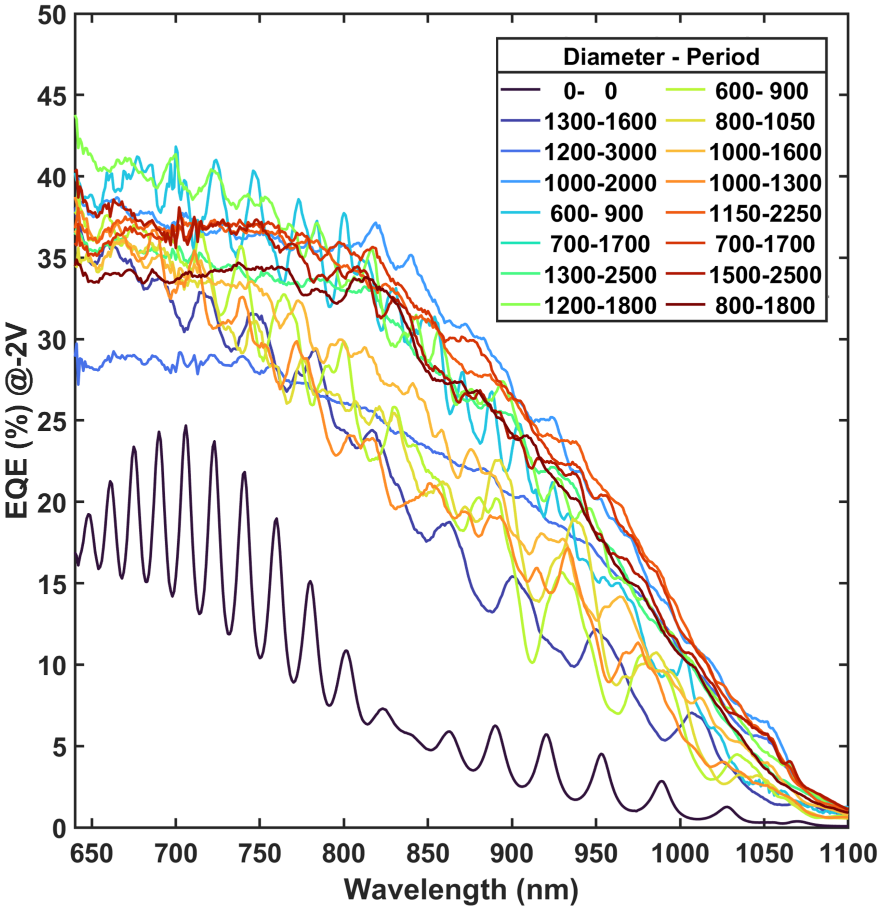

Fig. S8: **Measured unique absorption spectra:** EQE spectrum of individual unique detectors graphed for a range of PTST dimensions for a range of illumination wavelengths varying from 640-1100 nm at a fixed illumination power of 10 µW. The EQE is extracted at a fixed 2 V reverse bias.

At first, we obtained baseline responsivity measurement data for each photodiode. Further, we synthetically generate spectra and calculate photocurrent using the responsivity

data and train the computational model. The peak wavelength of the spectra has been varied from 640-1100 nm with a full-width half maxima ranging from 40-100 nm in our training sample. Finally, for a given respective set of photocurrents, we predict the unknown spectrum.

Mathematical formulation

For a given $F(\lambda)$ input spectral signal the photocurrent in each of the 16 photodiodes can be formulated as:

$$I_i = \int_{\lambda_0}^{\lambda_{end}} F(\lambda) R_i(\lambda)\, d\lambda \tag{S1}$$

For $i = 1, 2, ..., M$.

where $R_i(\lambda)$ represents the spectral response of the ith photodiode in the array. To reconstruct the input spectral signal $F(\lambda)$, we discretize the wavelength λ as $\lambda_j = \lambda_0 + j\Delta\lambda$, where, $j = 0, 1, , N$. The discretized equation S1 can be transformed as:

$$y = Rf + n \tag{S2}$$

where $y = [I_1, I_2, ..., I_M]^T$, $f = [F(\lambda_0), F(\lambda_1), ..., F(\lambda_N)]^T$, $R_{ij} = R_i(\lambda_(j-1))$, $i = 1, 2, ..., M$, $j = 1, 2, ..., N+1$, and n is noise. In our discretized formulation, the noise components are neglected, i.e., $n = 0$ and $\Delta F(\lambda) = 0$.

Since $N > M$, i.e., there are more unknowns than the number of equations. Therefore, the task of solving for f using equation S2 becomes underdetermined.[14] We have addressed this challenge by formulating a linear combination approximation method.

We implement this method by considering a set of basis spectra $F_T(\lambda)$ (Gaussian or Sine waves) of different spectral widths varying from 1 nm to 100 nm. Using the kth basis spectra as input, the photocurrent I_{Tk} at each photodiode can be calculated using the following

equation:

$$I_{Tk_i} = \int_{\lambda_0}^{\lambda_{end}} F_{Tk}(\lambda) R_i(\lambda)\, d\lambda \tag{S3}$$

For $i = 1, 2, , M,\ k = 1, 2, , P$. where $R_i(\lambda)$ represents the spectral response of the ith photodiode in the array and $F_T(\lambda) = [F_{T1}(\lambda), F_{T2}(\lambda), ..., F_{TP}(\lambda)]$ is the known set of basis spectra. The equation S3 can be discretized as:

$$y_T = Rf_T \tag{S4}$$

where $y_{Tik} = [I_{1k}, I_{2k}, ..., I_{Mk}]^T$, $f_T = [F_{Tk}(\lambda_0), F_{Tk}(\lambda_1), ..., F_{Tk}(\lambda_N)]^T$, $R_{ij} = R_i(\lambda_{(j-1)})$, $i = 1, 2, ..., M,\ j = 1, 2, ..., N+1,\ k = 1, 2, ..., P$. Now, the basis spectra are selected such that a linear combination of the input spectral signal is achieved. A good estimation can be achieved using Gaussian or Sine waves in the spectral domain as the basis spectra.

$$f = cf_T \tag{S5}$$

where $c = [C_1, C_2, ..., C_P]$ is the coefficient of the linear combination, $f = [F(\lambda_j)]$, $f_T = [F_{T1}(\lambda_j), F_{T2}(\lambda_j), ..., F_{TP}(\lambda_j)]$, $j = 1, 2, ..., N+1$, as explained in equation S2 and equation S4.

Linear combination approximation suggests that if the intensity of the spectral input can be estimated using the coefficients, c then the photocurrents can be represented using the same set of coefficients.[15,16]

$$y_T = Rf_T \Rightarrow c = \frac{y}{y_T} \tag{S6}$$

Using equation S6 we can obtain the linear coefficients, c; these coefficients are then used in equation S5 to estimate the input spectral signal, y. Since y_T is not a square matrix, we use a pseudo-inversion of the matrix to obtain the solutions to the coefficients. The choice of the basis spectra is further optimized by training against a known set of synthetic data

to improve accuracy.

Simulated result

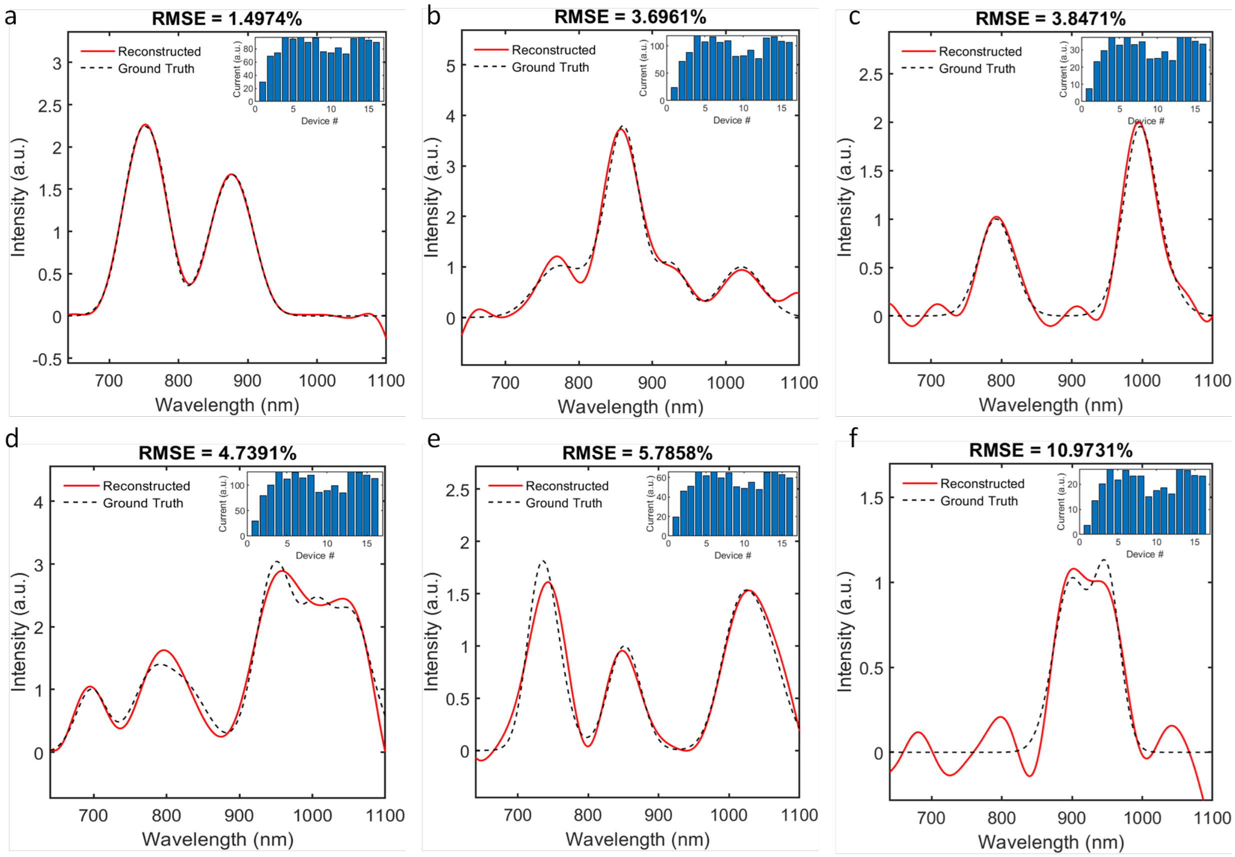

Fig. S9: **a-f**, **Reconstructed spectrum:** Extensive testing of the reconstructed spectra for a variety of ground truth signals. The black dashed line represents the original signal, i.e., the ground truth. The red solid line represents the reconstructed signal. The insets show the photocurrent profiles detected by the 16 photodiodes corresponding to each ground truth. Based on the input spectral signal, the accuracy of the reconstruction measured in terms of root mean squared error (RMSE), varies from 1.49% to 10.97%.

Fig. S9 presents the predicted spectra corresponding to a given ground truth. In our current formulation, the accuracy depends on the nature of the input signal, and the root mean squared error (RMSE) in reconstruction is shown to vary from 1.49% to 10.97%.

References

(1) Rawat, A.; Ahamed, A.; Bartolo-Perez, C.; Mayet, A. S.; McPhillips, L. N.; Islam, M. S. Design and Fabrication of High-Efficiency, Low-Power, and Low-Leakage Si-Avalanche Photodiodes for Low-Light Sensing. *ACS Photonics* **2023**, *10*, 1416–1423, http://dx.doi.org/10.1021/acsphotonics.3c00026.

(2) Yablonovitch, E. Photonic Crystals. *Journal of Modern Optics* **1994**, *41*, 173–194, http://dx.doi.org/10.1080/09500349414550261.

(3) Minin, I.; Minin, O. *Diffractive Optics and Nanophotonics: Resolution Below the Diffraction Limit*; Springer.

(4) Yu, N.; Capasso, F. Optical Metasurfaces and Prospect of Their Applications Including Fiber Optics. *J. Lightwave Technol.* **2015**, *33*, 2344–2358, https://opg.optica.org/jlt/abstract.cfm?URI=jlt-33-12-2344.

(5) Stefanov, K. D.; Dryer, B. J.; Hall, D. J.; Holland, A. D.; Pratlong, J.; Fryer, M.; Pike, A. A global shutter CMOS image sensor for hyperspectral imaging. SPIE Proceedings. 2015; https://doi.org/10.1117/12.2187856.

(6) Altaqui, A.; Sen, P.; Schrickx, H.; Rech, J.; Lee, J.-W.; Escuti, M.; You, W.; Kim, B. J.; Kolbas, R.; O'Connor, B. T.; Kudenov, M. Mantis shrimp–inspired organic photodetector for simultaneous hyperspectral and polarimetric imaging. *Science Advances* **2021**, *7*, https://doi.org/10.1126/sciadv.abe3196.

(7) Bao, J.; Bawendi, M. G. A colloidal quantum dot spectrometer. *Nature* **2015**, *523*, 67–70, http://dx.doi.org/10.1038/nature14576.

(8) Altaqui, A.; Kolbas, R. M.; Escuti, M. J.; O'Connor, B. T.; Kudenov, M. W. Organic-based photodetectors for multiband spectral imaging. *Applied Optics* **2021**, *60*, 2314, https://doi.org/10.1364/ao.417069.

(9) Grotevent, M. J.; Yakunin, S.; Bachmann, D.; Romero, C.; de Aldana, J. R. V.; Madi, M.; Calame, M.; Kovalenko, M. V.; Shorubalko, I. Integrated photodetectors for compact Fourier-transform waveguide spectrometers. *Nature Photonics* **2022**, *17*, 59–64, https://doi.org/10.1038/s41566-022-01088-7.

(10) Ennis, R.; Schiller, F.; Toscani, M.; Gegenfurtner, K. R. Hyperspectral database of fruits and vegetables. *Journal of the Optical Society of America A* **2018**, *35*, B256, http://dx.doi.org/10.1364/JOSAA.35.00B256.

(11) Stuart; McGonigle; Willmott Hyperspectral Imaging in Environmental Monitoring: A Review of Recent Developments and Technological Advances in Compact Field Deployable Systems. *Sensors* **2019**, *19*, 3071, http://dx.doi.org/10.3390/s19143071.

(12) Maldonado, A. I. L., Fuentes, H. R., Contreras, J. A. V., Eds. *Hyperspectral Imaging in Agriculture, Food and Environment*; InTech, 2018; https://doi.org/10.5772/intechopen.70213.

(13) Liu, J. et al. A near-infrared colloidal quantum dot imager with monolithically integrated readout circuitry. *Nature Electronics* **2022**, *5*, 443–451, http://dx.doi.org/10.1038/s41928-022-00779-x.

(14) Ahamed, A.; Wang, W.; Rawat, A.; McPhillips, L. N.; Ponizovskaya Devine, E.; Wang, S.-Y.; Ding, Z.; Islam, M. S. Advancing multi-dimensional vision: AI-driven imaging using unique photodetectors with integrated surface nanostructures. Low-Dimensional Materials and Devices 2023. 2023; http://dx.doi.org/10.1117/12.2682104.

(15) Ahamed, A.; Rawat, A.; McPhillips, L. N.; Marcu, L.; Islam, M. S. On-chip hyperspectral detectors for fluorescence lifetime imaging. High-Speed Biomedical Imaging and Spectroscopy IX. 2024; http://dx.doi.org/10.1117/12.3002476.

(16) Ahamad, A.; Ghandiparsi, S.; Bartolo-Perez, C.; Mayet, A. S.; Cansizoglu, H.; Devine, E. P.; Elrefaie, A. F.; Dhar, N. K.; Wang, S.-Y.; Yang, W.; Islam, M. S. Smart nanophotonics silicon spectrometer array for hyperspectral imaging. Conference on Lasers and Electro-Optics. 2020; http://dx.doi.org/10.1364/CLEO_SI.2020.STh3M.2.
